# Accurate quantification of nascent and mature RNAs from single-cell and single-nucleus RNA-seq

**DOI:** 10.1093/nar/gkae1137

**Published:** 2024-12-06

**Authors:** Delaney K Sullivan, Kristján Eldjárn Hjörleifsson, Nikhila P Swarna, Conrad Oakes, Guillaume Holley, Páll Melsted, Lior Pachter

**Affiliations:** Division of Biology and Biological Engineering, California Institute of Technology, 1200 E California Blvd, Pasadena, CA 91125, USA; UCLA-Caltech Medical Scientist Training Program, David Geffen School of Medicine, University of California, Los Angeles, 885 Tiverton Drive, Los Angeles, CA 90095, USA; Department of Computing and Mathematical Sciences, California Institute of Technology, 1200 E California Blvd, Pasadena, CA 91125, USA; Division of Biology and Biological Engineering, California Institute of Technology, 1200 E California Blvd, Pasadena, CA 91125, USA; Division of Biology and Biological Engineering, California Institute of Technology, 1200 E California Blvd, Pasadena, CA 91125, USA; deCODE Genetics/Amgen Inc., Sturlugata 8, 101 Reykjavík, Iceland; deCODE Genetics/Amgen Inc., Sturlugata 8, 101 Reykjavík, Iceland; School of Engineering and Natural Sciences, University of Iceland, Sæmundargata 2, 102 Reykjavík, Iceland; Division of Biology and Biological Engineering, California Institute of Technology, 1200 E California Blvd, Pasadena, CA 91125, USA; Department of Computing and Mathematical Sciences, California Institute of Technology, 1200 E California Blvd, Pasadena, CA 91125, USA

## Abstract

In single-cell and single-nucleus RNA sequencing (RNA-seq), the coexistence of nascent (unprocessed) and mature (processed) messenger RNA (mRNA) poses challenges in accurate read mapping and the interpretation of count matrices. The traditional transcriptome reference, defining the “region of interest” in bulk RNA-seq, restricts its focus to mature mRNA transcripts. This restriction leads to two problems: reads originating outside of the “region of interest” are prone to mismapping within this region, and additionally, such external reads cannot be matched to specific transcript targets. Expanding the “region of interest” to encompass both nascent and mature mRNA transcript targets provides a more comprehensive framework for RNA-seq analysis. Here, we introduce the concept of distinguishing flanking *k*-mers (DFKs) to improve mapping of sequencing reads. We have developed an algorithm to identify DFKs, which serve as a sophisticated “background filter”, enhancing the accuracy of mRNA quantification. This dual strategy of an expanded region of interest coupled with the use of DFKs enhances the precision in quantifying both mature and nascent mRNA molecules, as well as in delineating reads of ambiguous status.

## Graphical Abstract

**Graphical Abstract:**
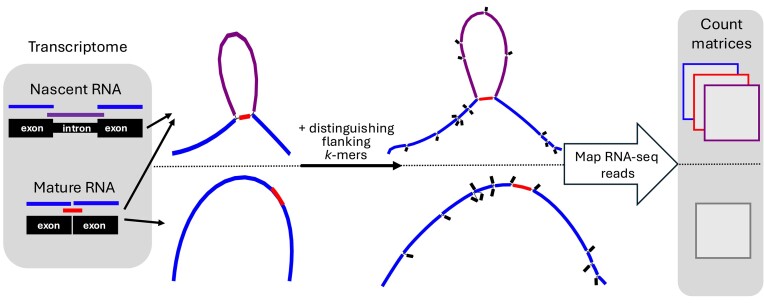

## Introduction

The utility of single-cell RNA sequencing (RNA-seq) measurements for defining cell types has represented a marked improvement over bulk RNA-seq, and has driven rapid development and adoption of single-cell RNA-seq assays (1). One application of single-cell RNA-seq that is not possible with bulk RNA-seq is the study of cell transitions and transcription dynamics, even via snapshot single-cell RNA-seq experiments (2–4). Such applications of single-cell RNA-seq are based on the quantification of both unprocessed and processed messenger RNAs (mRNAs) (Supplementary Figure S1), lending import to the computational problem of accurately and separately quantifying these two modalities (5). The importance of quantifying unprocessed mRNAs in addition to processed mRNAs has also been brought to the fore with single-nucleus RNA-seq (6–8).

The traditional approach in quantifying RNA-seq has been to rely on a reference transcriptome that defines a “region of interest”—typically restricted to mature mRNA transcripts (i.e. no introns) for bulk RNA-seq analyses. This conventional focus has been adequate for the broad objectives of bulk sequencing but is insufficient for the more granular and precise requirements of single-cell and single-nucleus RNA-seq. In these more detailed analyses, reads that emanate from outside the traditional “region of interest” present two problems: a high risk of mismapping within this defined region and the inability to be matched to specific targets within the transcriptome index.

**Figure 1.**
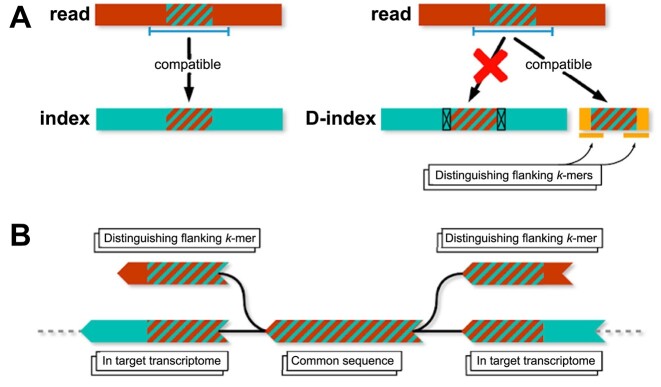
Overview of DFKs. (**A**) A nontranscriptomic read containing a subsequence of length greater than *k*, which also occurs in a transcript in the target transcriptome index, will get attributed to that transcript. DFKs, here shown in the modified index (the D-index), can be used to determine whether a read compatible with a reference transcriptome may have originated from elsewhere in the genome. In this diagram, the D-index is one constructed with DFKs. The hatched region depicts the *k*-mers shared between the read and the index, and the line underneath the read shows the sequence stretch spanning both those shared *k*-mers plus the DFKs. (**B**) A dBG representation of DFKs.

To address the problem of mismapping of external reads (9), we introduce distinguishing flanking *k*-mers (DFKs) (Figure 1, Algorithm 1, “Materials and methods” section) to identify reads that are external to the sequences present in the transcriptome index. DFKs are a minimal set of *k*-mers that can be used to distinguish whether a read that is mapped to a set of targets in the transcriptome index has its origin from within the transcriptome index or has an external origin. These *k*-mers thus can act as a filter to prevent reads of external origin from being mismapped to the transcriptome index. In other words, these *k*-mers, if present in a read, will cause the read to be filtered out. We use the term D-list (distinguishing list) to denote the sequences from which DFKs are extracted based on the contents of the transcriptome index. By default, the D-list is set to the genome FASTA file. Therefore, hereinafter, specifying the usage of a D-list refers to supplying the genome FASTA file as the D-list. While using standard mature mRNA transcriptome index with a D-list can be used to improve the quantification of single-cell RNA-seq due to intronic and intergenic reads, still, only mRNA transcripts exist in the index and hence, only reads mapping to mature mRNA regions will be considered. While this is useful for certain applications of single-cell analyses such as cell type identification, having only a single-cell count matrix prevents the usage of biophysical models which jointly consider mature and nascent RNA quantifications (10,11). Thus, extending the index (5,12,13) to allow quantifications of RNA molecules at different stages of their processing is important, as will be discussed next.

**Figure 2.**
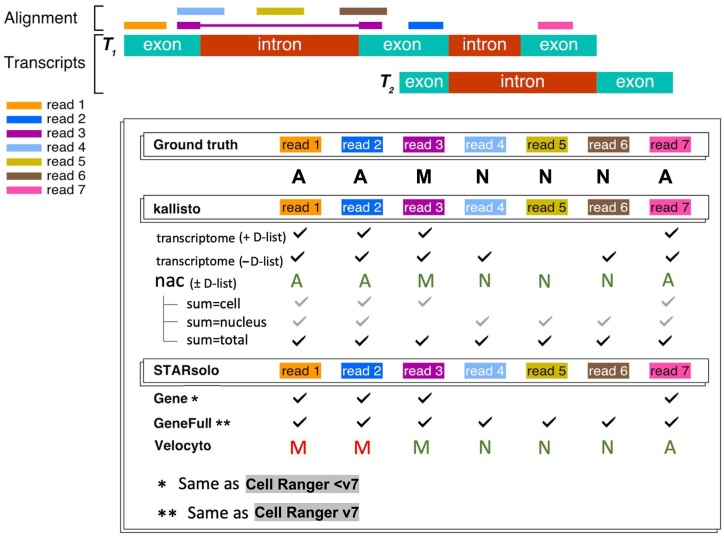
Approach to read assignment and classification into nascent, mature and ambiguous categories by kallisto, STARsolo and Cell Ranger. This classification of reads enables accurate classification of RNA species, enabling ambiguous (A) reads to be assigned in various ways based on context [e.g. ambiguous reads are allocated to “mature” (M) in single-cell RNA-seq splicing analysis or added to both “mature” (M) and “nascent” (N) in the case of quantifying “total” RNA content]. The kallisto nac index in this example produces the same classification with or without the D-list because no external reads (i.e. those existing outside annotated genomic regions) are present. The standard “transcriptome” index cannot resolve different RNA species and, without the D-list, will result in some reads originating from nascent transcripts (i.e. reads cross exon–intron boundaries) being mapped even though introns do not exist in the index; however, with the D-list, those intronic *k*-mers in the reads will map to DFKs in the index. The --sum options for the nac index represent the various ways the N, M and A matrices can be summed up (i.e. using M + A for “cell” and N + A for “nucleus” or N + A + M for “total”). Results for alevin-fry are not shown because its *spliceu* index produces classifications identical to kallisto. The checkmarks represent whether a given read will be counted and the letters M, N and A represent the read classifications (with red letters denoting classifications that differ from the ground truth).

To quantify nascent RNA transcripts, it is necessary to extend the transcriptome index to include such targets. That is, an index should be created that encompasses the nascent RNAs and the mature RNAs. While seemingly straightforward to construct such an index and to map reads against it, a difficulty arises from classifying individual reads, or individual unique molecular identifiers (UMIs), as being of “mature” or “nascent” status. This difficulty stems from the fact that sequenced reads are typically much shorter than transcripts, and therefore there can be ambiguity in classification of reads as “mature” or “nascent”. Reads that span an exon–exon junction must originate from a completely or partially processed mRNA (which we call “mature”), whereas reads containing sequence unique to an intron must originate from a completely unprocessed or partially processed mRNA (which we call “nascent”). However there are many reads for which it is impossible to know whether they originated from an unprocessed or processed transcript (hence, are ambiguous) (Figure 2). Methods that rely on *k*-mer mapping must account for the distinction between *k*-mer ambiguity and read ambiguity, and this distinction has not been carefully accounted for in previous *k*-mer based single-cell RNA-seq pre-processing workflows (12,13).

To classify reads as nascent, mature or ambiguous, we first pseudoalign reads using kallisto (14) against a kallisto index containing the mature mRNA (as used originally in pseudoalignment) and nascent mRNA. The nascent mRNA spans the full length of a gene and contains both the gene’s exons and introns as a single contiguous sequence. This comprehensive representation, implemented as the nac index in kallisto (15), allows for accurate classification of reads as mature or nascent or ambiguous, as it properly accounts for the exon–intron boundary and acknowledges that exons are components of both nascent and mature mRNA (Figure 2). The developers of alevin-fry have also adopted this approach in the alevin-fry *spliceu* index (16), an updated version of the original alevin-fry *splici* index. However, other tools, such as STARsolo (9) and the popular Cell Ranger software (17), do not produce such classifications.

While the nac index contains both mature and nascent mRNA, reads of external origin could still arise from intergenic regions of the genome being sequenced. These reads may still be erroneously mapped to this extended transcriptome index. To mitigate the possibility of such instances occurring, one would want to use DFKs by using the nac index with a D-list. This approach is implemented in kallisto by default when building the nac index. Altogether, the nac index, in conjunction with DFKs to mask out reads of external origin, enables the accurate quantification and classification of nascent, mature and ambiguous mRNA.

## Materials and methods

### The D-list

A D-list enables accurate quantification of RNA-seq reads in experiments where reads that are not an expression of the target transcriptome may still contain sequences which do occur in the target transcriptome. Without the D-list, these reads may be erroneously quantified as transcripts in the target transcriptome, based on alignment of the common sequences. Thus, the D-list may contain any sequences that are not desired in the abundance matrix yielded by the quantification. Such sequences may include genomes of other organisms (18) to avoid mismapping due to sample contamination, they may consist of the genome from which the target transcriptome was made, or they may contain common transposable elements, such as Alu regions, which might confound analyses. The D-list is incorporated into the index by finding all sequences, *k* base-pairs or longer, that occur in both the D-list and the target transcriptome. The first *k*-mer upstream and the first *k*-mer downstream of each such common sequence in the D-list are added to the index-colored *de Bruijn* graph (dBG). We refer to these new vertices in the graph as DFKs (Algorithm 1). The DFK vertices are left uncolored in the index, such that during quantification, reads that contain them will be masked out, and go unaligned.

As an illustration, consider a read containing both *k*-mers found only in intergenic RNA and *k*-mers found both in mRNA and the intergenic RNA. If that read is mapped to an index built from mRNA transcripts, the mRNA *k*-mers will be found in the index, whereas the disambiguating genome *k*-mers will not. The whole read will be erroneously mapped based on the ambiguous *k*-mers (i.e. the *k*-mers found both in mRNA and the intergenic RNA) that are present in the index. By finding all ambiguous *k*-mers in the mRNA index, and adding any distinguishing flanking genome *k*-mers to the index, the read will be masked from mapping to an mRNA transcript.

Recent papers have discussed various ways of reducing the number of false positives in RNA quantification through either including the entire genome or a subset of the genome in the index as a “decoy” or through alignment scoring (19). Our method is distinct in that it incorporates only the minimum amount of data, required to disambiguate common sequences, into the index while still adhering strictly to the principles of *k*-mer based pseudoalignment. Therefore, the memory usage and runtime of using pseudoalignment using a D-list are on par with the memory usage and runtime without the use of a D-list. Our method can scale favorably to larger genome size (or, more generally, larger D-lists) while the target sequences to map against remain small.

The D-list is implemented in kallisto version 0.50.1 (14,15). The kallisto index command contains a --d-list option that takes in, as an argument, the path to a FASTA file containing the D-list sequences for building an index with the D-list. The kallisto index command also contains a --d-list-overhang option for specifying longer overhangs (i.e. extending the flanking sequences that make up the DFKs). The kallisto bus command (20) contains a --dfk-onlist option that, when enabled, adds a D-list target to the equivalence class (EC) for a given pseudoalignment if a DFK is encountered rather than discards the read; this option is useful for distinguishing reads that do not pseudoalign versus reads that are discarded due to a DFK. Finally, in kb-python (version 0.28.0), the kb ref command automatically uses the genome FASTA as the D-list when building the kallisto index – a behavior that can be overwritten by explicitly specifying --d-list in kb ref. As a minor nuance, the default genome FASTA D-list does not contain splice junctions (SJs); however, the number of additional DFKs that would be indexed with the inclusion of SJ-spanning sequences is miniscule since SJ-spanning contigs are only *k* − 1 number of *k*-mers in length. Therefore, including the spliced transcriptome in the D-list would be unlikely to make any difference in read mapping.

### Generating DFKs

A sequence *s* is a string of symbols drawn from an alphabet Σ = {A, T, C, G}. The length of *s* is denoted by |*s*|. A substring of *s* is a string that occurs in *s*: it has (zero-indexed) start position *i* and end position *j* and is denoted by *s*[*i* : *j*], therefore |*s*[*i* : *j*]| is equal to *j* − *i*. In the case that |*s*[*i* : *j*]| equals *k*-mer size *k*, *s*[*i* : *j*] is *k*-mer. A compact *de Bruijn* graph (cdBG) is a dBG where all maximal nonbranching paths of vertices from a dBG, wherein each vertex is a *k*-mer, are merged into single vertices (21). Each vertex in a cdBG is a sequence called a unitig. We define a cdBG *U* as a set where each element *u* ∈ *U* is a unitig. The function Map(*s, u*) takes in a *k*-mer *s* and a unitig *u*, and returns the position of *s* along *u* if *s* exists in *u*, or NULL otherwise. Algorithm 1 applies these definitions toward identifying *DFKs* from a D-list *D*, given a cdBG *U* of *k*-mer size *k* built over target sequences (e.g. a transcriptome). For expository purposes, the algorithm is described such that *U* is a nonbidirected cdBG (i.e. the *k*-mers and their reverse complements are *not* represented identically). However, in practice each *k*-mer and its reverse complement are represented as a single canonical *k*-mer (the lexicographic minimum of the *k*-mer and its reverse complement). Additionally, for simplicity, we define DFKs and describe the algorithm only for single overhangs.


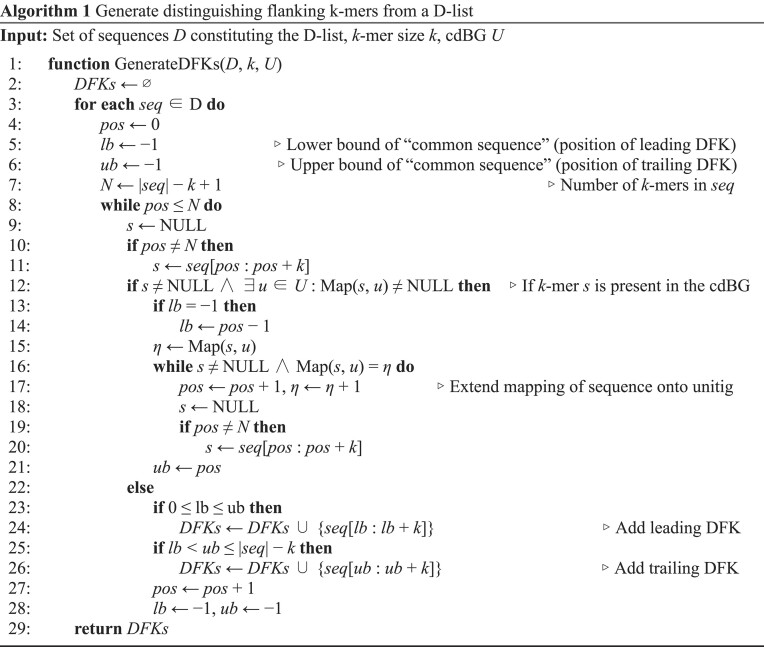


Next, we discuss the space complexity of DFKs.

**Lemma 1**. The worst case space complexity of DFKs is O(min(*N_k_*,*M_k_*)) where *N_k_*is the number of unique *k*-mers in the dBG and *M_k_* is the number of unique *k*-mers in the D-list.

**Proof**. Considering the alphabet Σ = {A,T,C,G}, ∀s ∈ dBG, the maximum number of flanking *k*-mers on each side of *s* is |Σ|, permitting a flanking *k*-mer for each character in the alphabet. On each side of *s*, the maximum number of DFKs, which are the flanking *k*-mers in the D-list but not in the dBG, is |Σ| − 1 corresponding to the presence of one flanking *k*-mer that exists in the dBG and the remaining |Σ| − 1 *k*-mers being DFKs. Since *s* has two sides (leading and trailing), the maximum number of DFKs becomes 2(|Σ| − 1) = 6. In the worst-case scenario, ∀s ∈ dBG, *s* contains the maximum number of DFKs. Thus, |DFKs| ≤ 6*N_k_* where |DFKs| is the cardinality of the set of DFKs. The actual number of DFKs identified from the D-list is bounded by the number of unique *k*-mers in the D-list, denoted as *M_k_*, i.e. |DFKs| ≤ *M_k_*. Since |DFKs| ≤ min(6*N_k_*, *M_k_*), the space complexity for storing DFKs is O(min(*N_k_*, *M_k_*)).

### Improvements to the kallisto index

The dBG implementation in kallisto was replaced with Bifrost (21), which employs a minimizer (22) lookup table in lieu of a *k*-mer lookup table in order to achieve a lower memory footprint. Furthermore, since the set of minimizers in the graph is known at the time of quantification, we replaced the minimizer hash function with BBHash (23), which implements a minimal perfect hash function. This enables kallisto to shrink the minimizer hash table to capacity, saving memory. Additionally, ECs were restructured. Sets of transcripts are represented as Roaring bitmaps (24) and a Robin Hood hashmap (25) is used for the inverted hash table mapping transcript sets to EC. ECs are allocated dynamically during quantification. Thus, an EC is only created once it has been found to be used by a read, whereas the previous paradigm preemptively created the ECs used by all the vertices in the graph, during indexing, regardless of whether or not they were ever used by a read. Finally, the index includes an external Robin Hood hashmap for storing DFKs since, as the number of DFKs is relatively small, the external hashmap occupies less memory, with only a small reduction in speed, compared with integrating the DFKs into the main dBG (Supplementary Figure S2). These changes have resulted in an approximately 2× reduction in runtime and 4× reduction in memory consumption in kallisto v0.50.1 compared with kallisto version 0.48.0 when using the nac index type to map single-cell RNA-seq reads (Supplementary Figure S2).

### Simulation frameworks

We obtained the simulation framework developed by the authors of STARsolo (9) from https://github.com/dobinlab/STARsoloManuscript/ and ran the simulation as-is to generate a ground truth matrix. For the kallisto nac index type, the “mature” and “ambiguous” count matrices were summed up by using --sum=cell in kb count and the resultant matrix was used for testing. For all tools, a predefined “on list” of barcodes (referred to in other tools as a whitelist or an unfiltered permit list) was supplied. The three simulated sequencing datasets used are as follows:

- No multigene: 339 million reads;
- With multigene: 350 million reads; and
- Exon-only, no multigene: 189 million reads.

For the simulations where errors were introduced into sequences, the reads were processed to first introduce mismatches, then introduce deletions and then introduce insertions.

For the bulk RNA-seq simulations, RSEM (26) was used to generate 20 sets of 30 million 75-bp paired-end simulated reads following the methods described in the original kallisto publication. The “ground truth” transcript-level estimated counts were compared with those produced by kallisto. Note that only the transcripts associated with 25% of genes, selected at random, were indexed by kallisto. We chose to select 25% of genes to represent an incomplete transcriptome.

### Runtime and memory usage assessment

The command /usr/bin/time -v which executes the GNU time program was used to obtain the elapsed (wall clock) time and the maximum resident set size for runtime and peak memory usage, respectively. All performance assessments were conducted on a server with x86-64 architecture, 88 CPUs (Intel Xeon Gold 6152 CPU @ 2.10GHz) and 768 GB of memory.

### Evaluation metrics for single-cell RNA-seq simulations

To evaluate the performance of a program’s output gene count matrix ${{{\boldsymbol{G}}}_{\boldsymbol{p}}} \in {{\mathbb{R}}^{{\boldsymbol{\ n}} \times {\boldsymbol{m}}}}$ against a simulation’s ground truth gene count matrix ${{{\boldsymbol{G}}}_{\boldsymbol{s}}} \in {{\mathbb{R}}^{{\boldsymbol{\ n}} \times {\boldsymbol{m}}}}$, where $n$ is the number of cells, $m$ is the number of genes, ${{\hat{y}}_{ij}}$ is the count of gene $j$ in cell $i$ in ${{{\boldsymbol{G}}}_{\boldsymbol{p}}}$ and ${{y}_{ij}}$ is the count of gene $j$ in cell $i$ in ${{{\boldsymbol{G}}}_{\boldsymbol{s}}}$, the following metrics are used:

Root mean squared error (RMSE):


\begin{equation*}RMSE = \sqrt {\frac{1}{{nm}} \sum\limits_{i = 1}^n {\sum\limits_{j = 1}^m {{{{({{y}_{ij}} - {{{\hat{y}}}_{ij}})}}^2}}}} \end{equation*}

False positive representation (FPR):


\begin{equation*}FPR = \frac{1}{{nm}}\sum\limits_{i = 1}^n {\sum\limits_{j = 1}^m {\left\{ {\begin{array}{@{}*{1}{c}@{}} {1\quad \text{if}\quad {{y}_{ij}} = 0\ \text{and}\ {{{\hat{y}}}_{ij}}\ >\ 0}\\ {0\quad \text{otherwise}\quad\quad\quad\quad\quad\quad} \end{array}} \right.} }\end{equation*}

False negative representation (FNR):


\begin{equation*}FNR = \frac{1}{{nm}}\sum\limits_{i = 1}^n {\sum\limits_{j = 1}^m {\left\{ {\begin{array}{@{}*{1}{c}@{}} {1\quad \text{if}\quad {{y}_{ij}}\ >\ 0\ \text{and}\ {{{\hat{y}}}_{ij}} = 0}\\ {0\quad \text{otherwise}\quad\quad\quad\quad\quad\quad} \end{array}} \right.} } \end{equation*}

Note that the FPR and FNR are defined such that the denominator is the total size of the matrix and therefore differ from the traditional false positive rate and false negative rate calculations.

Correlation coefficient: We use two per-cell correlation coefficients to assess the correlation between the “ground truth” simulated gene counts cell and the program’s output gene counts for a given cell. The first, *r*, is the Pearson correlation computed across all genes within a given cell. The second, ρ*, is a modified variant of the Spearman correlation in that the Spearman correlation is computed only using the genes that have a nonzero count in both the simulation and the program output within a given cell. This variant is the assessment used by the developers of the STARsolo simulations (9). The restriction to nonzero cells is necessary when using the Spearman correlation, as the zeroes cannot be ranked with respect to each other. However, we note that use of the Spearman (and therefore ignoring the zeroes) provides an assessment that is highly sensitive to low counts, especially the difference in a program reporting a one or a zero for a gene in a cell.

Pearson correlation for cell $i$, using all genes:


\begin{equation*}{{r}_i} = {\mathrm{pearson}}\left( {\left\{ {\left( {{{y}_{ij}},{{{\hat{y}}}_{ij}}} \right),\,\ j = 1, \cdots ,m} \right\}} \right)\end{equation*}

Spearman correlation for cell $i$, using only genes with a nonzero count in both the simulation and the program output for that cell:


\begin{equation*}{{\rho }_i}* = {\mathrm{spearman}}\left( {\left\{ {\left( {{{y}_{ij}},{{{\hat{y}}}_{ij}}} \right){\mathrm{|}}\left( {{{y}_{ij}},{{{\hat{y}}}_{ij}}} \right)\neq \left( {0,0} \right),\,\ j = 1, \cdots ,m} \right\}} \right)\end{equation*}

### Processing of SPLiT-seq data

Cell barcodes corresponding to C2C12 myoblast cells with at least 10 000 UMIs were extracted based on metadata obtained from the study which produced that dataset (27). The reads containing those barcodes were divided into oligo-dT reads and random hexamer reads based on the first round barcode sequence. These initial steps were performed using the splitcode program (28). Next, to mitigate spurious read alignment to low complexity intronic sequences, bowtie2 (29) was used to align the reads to an index of ribosomal RNAs, transfer RNAs, microRNAs and repetitive elements, and those reads were removed with seqkit (30). STAR (31) was used to align reads to the mouse reference genome to generate a BAM file, which was indexed with SAMtools (32) and visualized with Integrative Genomics Viewer (33). kallisto | bustools was used to pseudoalign reads to the mouse nac index (with D-list) and to produce transcript compatibility counts (TCCs). Normalized counts were produced by CP10k normalization followed by log1p transformation and processed with Scanpy (34).

### Assessment of count matrices for cell clustering and marker gene identification

Analysis of count matrices were performed following methods described in other work, which should be referred to for a more detailed description (35). Briefly, after filtering the count matrix for minimum three cells per gene, minimum 200 genes per cell and maximum 20% mitochondrial gene content, count data were CP10k normalized then log1p transformed. Highly variable genes were selected for, then normalized gene counts were scaled to zero mean and unit variance. Nearest neighbor graphs were constructed from the cell coordinates on the top 50 principal component analysis (PCA) embeddings. Clusters were formed from the Leiden algorithm (36) and then visualized on alluvial plots and Uniform Manifold Approximation and Projection (UMAP) plots (37,38). These processing steps, as well as selection of significant (adjusted *P*-value <0.05) marker genes across all clusters, were performed using Scanpy (34).

### Code availability

kallisto is available under the BSD-2-Clause license and is available at https://github.com/pachterlab/kallisto. Code for the analyses performed for this paper is available at https://github.com/pachterlab/SHSOHMP_2024.

### Software versions

Unless stated otherwise, the software versions used are as follows: kallisto 0.50.1, bustools 0.43.2, kb-python 0.28.0, salmon 1.10.0, alevin-fry 0.8.2, simpleaf 0.15.1, Rsubread 2.12.3, Cell Ranger 7.0.1, STAR 2.7.9a, Bowtie2 2.5.3, seqkit 2.8.0, SAMtools 1.19.2, Scanpy 1.9.5 and splitcode 0.30.0. Additionally, Bandage version 0.8.1 (39) was used for rendering dBGs into ribbon-like representations.

## Results

### DFKs improve single-cell RNA-seq quantification based on simulations

**Figure 3.**
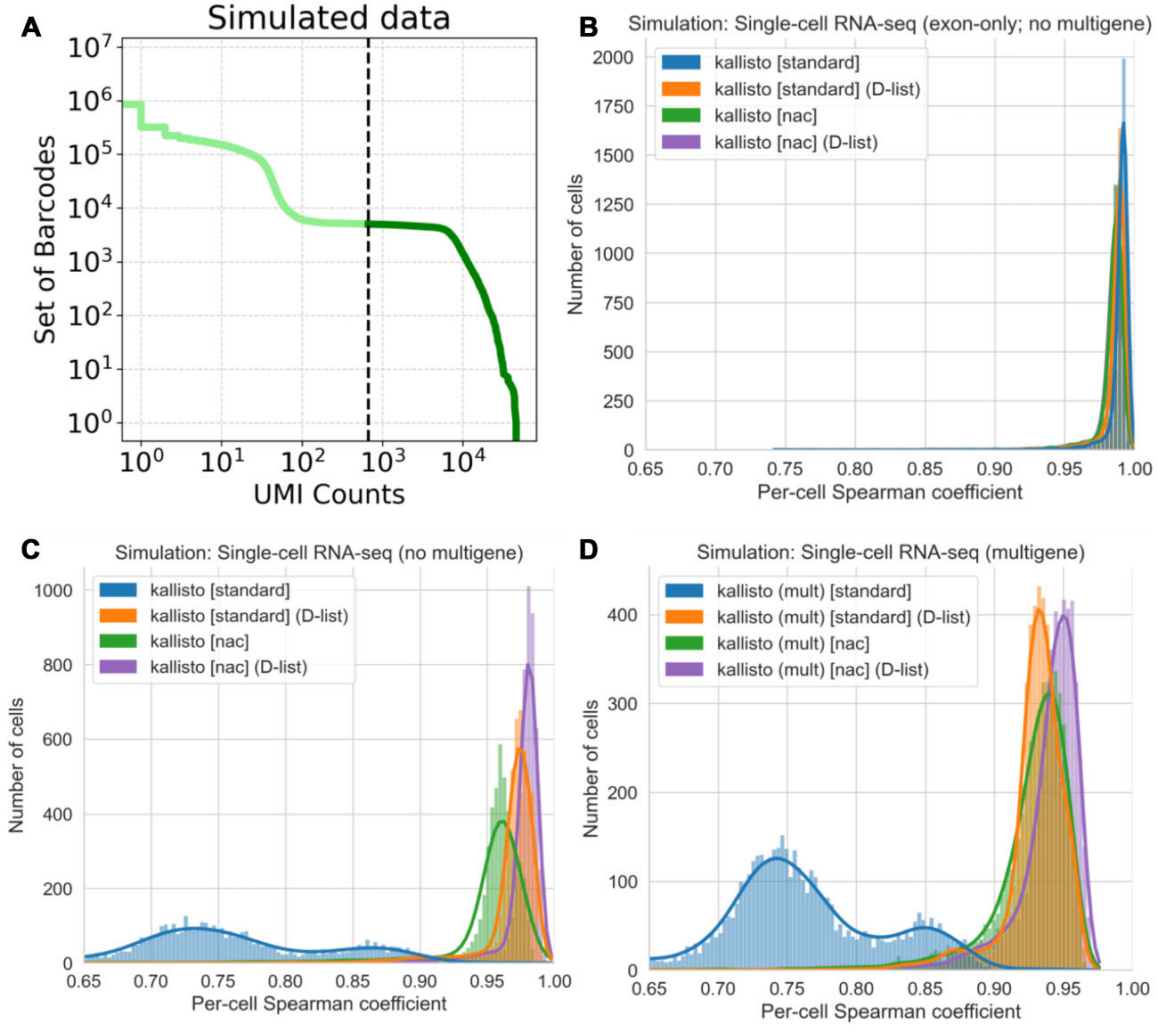
Assessment of the impact of DFKs on accuracy when tested on simulated data generated using the STARsolo simulation framework. (**A**) Knee plot of the truth count matrix from the STARsolo single-cell RNA-seq simulation. These simulated data represent the “no multigene” simulation. The 5000 cell barcodes with the highest UMI counts were filtered for (corresponding to a UMI threshold of 667). These 5000 cell barcodes were used in downstream analysis of all STARsolo simulated data. (**B**) Correlation between kallisto quantifications versus simulated truth for reads only spanning exons. (**C**) Correlation between kallisto quantifications versus simulated truth for single-cell RNA-seq reads that map to a single gene. (**D**) Correlation between kallisto quantifications versus simulated truth for single-cell RNA-seq reads that include multigene reads. mult: the multimapping quantification mode is enabled. The per-cell spearman correlation, ρ*, between gene counts was determined by excluding genes that contain zero counts in both the kallisto quantification and in the simulation quantification for a given cell barcode (see “Materials and methods” section).

**Figure 4.**
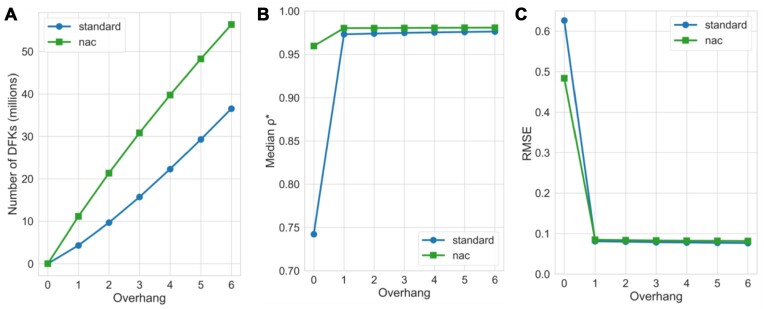
Assessment of the impact of longer overhang DFKs on accuracy when tested on simulated data generated using the STARsolo simulation framework. (**A**) The number of DFKs at various overhang settings. An overhang of 0 means no DFKs were used. An overhang of 1 is the default setting for the D-list implementation. (**B**) Median correlation coefficient ρ* (see “Materials and methods” section) between kallisto quantifications at various D-list overhang settings versus simulated truth for the “single-cell RNA-seq (no multigene)” simulation. (**C**) RMSE (see “Materials and methods” section) between kallisto quantifications at various D-list overhang settings versus simulated truth for the “single-cell RNA-seq (no multigene)” simulation.

To assess improvement of using pseudoalignment with DFKs on single-cell RNA-seq reads, we used the simulation framework developed by the authors of STARsolo (9). In that simulation framework, errors were introduced into reads at 0.5% mismatch error rate, and reads were simulated from both coding and noncoding genomic sequence to mimic the presence of both unprocessed, partially processed and completely processed transcripts in single-cell RNA-seq experiments. The top 5000 barcodes, based on UMI count from the simulated data, were used for analysis (Figure 3A). When quantifying simulated reads that only span exons with kallisto, the DFKs produced by a D-list do not considerably affect quantification accuracy (Figure 3B). However, upon including reads that span introns, the D-list improves the concordance between kallisto quantification count matrix and the simulated truth count matrix in both simulations that only include reads that map uniquely to one gene (Figure 3C) and in simulations that additionally include multigene reads (Figure 3D). The increase in accuracy from using a D-list is further evident in transcript-level estimates from bulk RNA-seq simulations when mapping reads against an incomplete transcriptome wherein reads from unindexed transcripts may affect mapping (Supplementary Figure S3). Interestingly, although the nac index type includes nascent and mature transcripts, the quantification accuracy still improves slightly with the use of a D-list, likely due to filtering out reads that originate from outside annotated genic loci. The evaluation metrics are shown in Table 1. Note that, for the nac index type, UMIs assigned to nascent transcripts were not used in the quantification because the simulation truth matrix does not include nascent transcript counts. For the multigene case, bustools was run with the multimapping option enabled when counting UMIs following kallisto quantification. Disabling this option resulted in slightly worse results, due to more false negatives, on the multigene simulation (Supplementary Figure S4). Enabling the multimapping mode had little effect when run on the exon-only simulations or the nonmultigene simulations (Supplementary Figure S5). While this mode can identify nonuniquely mapped reads by dividing UMI counts uniformly amongst the genes that the UMI is assigned to, it results in counts that are not whole numbers; thus, the standard for the field has been to discard such UMIs. Finally, since each DFK is only one *k*-mer flanking a unitig, we sought to assess whether considering more *k*-mers flanking a unitig as DFKs (i.e. longer overhangs) would improve the accuracy of kallisto (Figure 4A). We found that the benefit of including longer overhangs is negligible (Figure 4B and C; Supplementary Table S1); therefore, by default, we adhere to having exactly one DFK overhang.

**Table 1.** Evaluation metrics of kallisto on simulated data generated using the STARsolo simulation framework

Index type	D-list	mult	Median ρ*	Median *r*	RMSE	FPR	FNR	*k*-mers	DFKs
Simulation: single-cell RNA-seq (exon-only; no multigene)									
standard			0.991	0.999	0.04	0.00005	0.00036	113M	0
standard	✓		0.988	0.999	0.05	0.00004	0.00048	113M	4M
nac			0.986	0.999	0.06	0.00003	0.00059	1398M	0
nac	✓		0.985	0.999	0.06	0.00003	0.00061	1398M	11M
Simulation: single-cell RNA-seq (no multigene)									
standard			0.742	0.993	0.62	0.01940	0.00032	113M	0
standard	✓		0.973	0.999	0.08	0.00080	0.00048	113M	4M
nac			0.959	0.996	0.48	0.00064	0.00059	1398M	0
nac	✓		0.980	0.999	0.08	0.00020	0.00061	1398M	11M
Simulation: single-cell RNA-seq (multigene)									
standard		✓	0.751	0.991	0.66	0.05127	0.00026	113M	0
standard	✓	✓	0.932	0.999	0.16	0.00475	0.00048	113M	4M
nac		✓	0.933	0.995	0.52	0.03716	0.00056	1398M	0
nac	✓	✓	0.945	0.999	0.16	0.03240	0.00059	1398M	11M

mult: the multimapping quantification mode is enabled; ρ*: modified spearman correlation; *r*: Pearson correlation; see **“Materials and methods”** section for details.

### Assessment of other pseudoalignment and alignment based single-cell RNA-seq workflows on simulations

**Figure 5.**
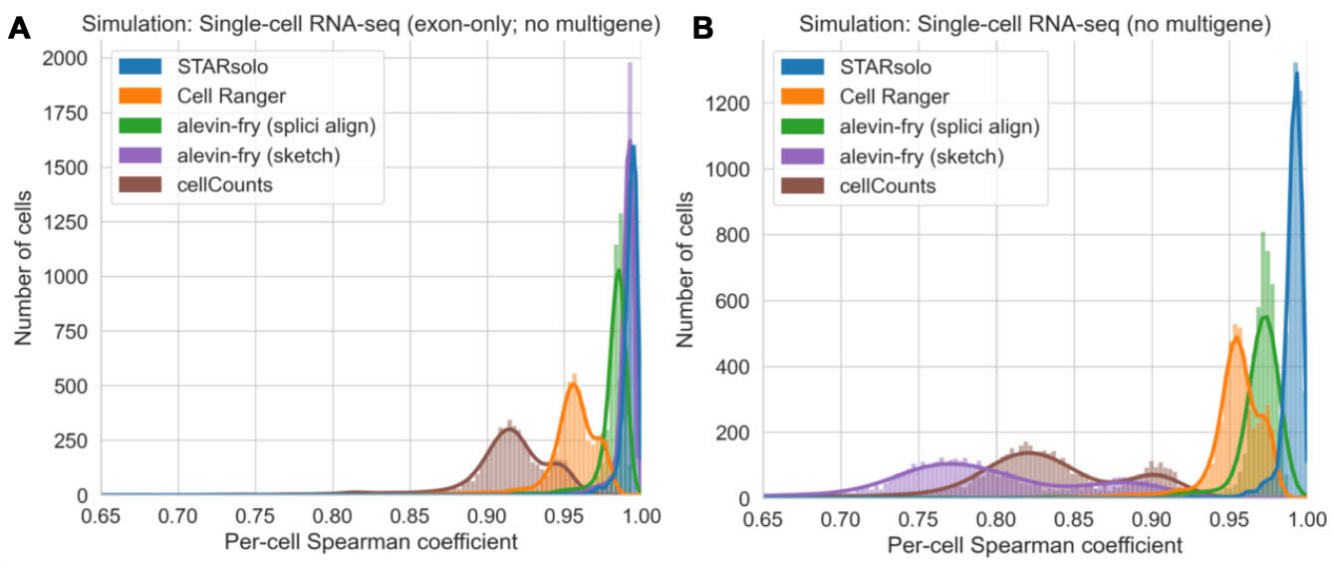
Assessment of different tools on simulated data generated using the STARsolo simulation framework. (**A**) Correlation between quantifications produced by the tools versus simulated truth for reads only spanning exons. (**B**) Correlation between quantifications produced by the tools versus simulated truth for single-cell RNA-seq reads that map to a single gene. Evaluation against multigene reads was not performed because of different methods exposed by different tools to handle such reads. The per-cell spearman correlation, ρ*, between gene counts was determined by excluding genes that contain zero counts in both the kallisto quantification and in the simulation quantification for a given cell barcode (see “Materials and methods” section). splici align: Enabling the index used by alevin-fry that contains introns as well as selective alignment mode. sketch: Selective alignment disabled and index is a standard transcriptome index that does not include introns in alevin-fry. For Cell Ranger, version 7 was used with the include-introns option set to false in order to mimic the default behavior of older versions of Cell Ranger. For cellCounts, the featureType option was set to “exon” (which is the default option) rather than “gene” in order to exclude intronic read quantification.

Next, we assessed the performance of other tools using the STARsolo simulation framework. Specifically, we assessed four tools: (i) STARsolo (9), a single-cell/nucleus RNA-seq tool built into the STAR aligner (31) program, (ii) Cell Ranger (17), the pipeline implemented by 10x Genomics, (iii) cellCounts(40), a tool based on the Rsubread aligner (41) and the featureCounts (42) program, and (iv) alevin-fry (13,43), a tool that leverages salmon (44) for pseudoalignment. We found that the tools produced quantifications that correlated well with the simulated ground truth for the simulated reads that only span exons (Figure 5A; Supplementary Table S2). However, on simulations including intronic reads, both alevin-fry, when executed in a standard pseudoalignment configuration against a spliced transcriptome, and cellCounts performed less well compared with STARsolo and Cell Ranger (Figure 5B; Supplementary Table S2). In the case of alevin-fry, using an expanded index that includes introns eliminated this decrease in performance, which is consistent with prior reports (13). Additionally, enabling selective alignment mode (19) in alevin-fry resulted in further accuracy improvements, similar to the improvements yielded by the D-list, even when used with an expanded transcriptome index. Note that the same simulated data were used in Supplementary Table S2 and Table 1, making the results directly comparable between kallisto (Table 1) and other software (Supplementary Table S2).

### DFKs incur an only minor increase in memory usage and runtime when mapping RNA-seq reads

**Figure 6.**
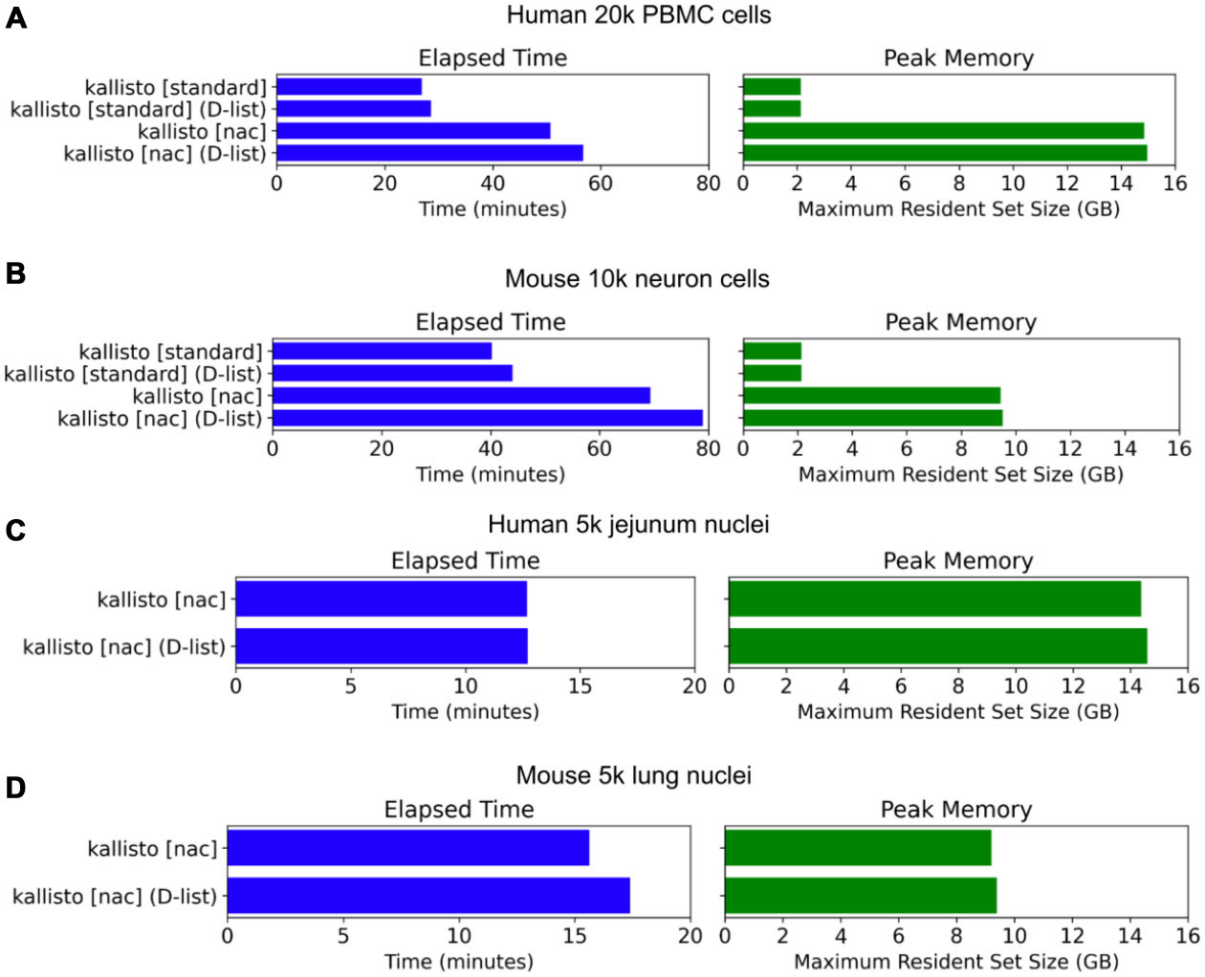
Runtime and memory usage of kallisto with different index types. (**A** and **B**) Runtime (on 16 threads) and memory usage of the standard index type and the nac index type, created with and without a D-list, on single-cell RNA-seq data generated with 10x Genomics. (**C** and **D**) Runtime (on 16 threads) and memory usage of the nac index type, created with and without a D-list, on single-nucleus RNA-seq data generated with 10x Genomics. The standard index type was not employed for single-nucleus RNA-seq data because single-nucleus RNA-seq reads predominantly originate from intron-containing pre-mRNA.

We assessed the impact of DFKs on memory usage and runtime when processing RNA-seq reads. Across single-cell and single-nucleus RNA-seq datasets from human and mouse tissue, DFKs resulted in only a minor increase in memory usage and runtime. Memory usage increased by <2% which is on the order of megabytes while runtime increased by <15% (Figure 6). On the other hand, mapping RNA-seq reads with the nac index type resulted in a much more substantial increase in memory usage and runtime compared with the standard index type. These results make sense as the nac index type is 10 times larger than (i.e. contains 10 times as many *k*-mers as) the standard index type, whereas the DFKs extracted from a D-list are only a small percentage (i.e. less than 5%) of the total number of *k*-mers. Thus, DFKs can substantially improve RNA-seq mapping accuracy without having a major impact on performance.

### DFKs maintain robustness to sequencing errors during mapping

**Figure 7.**
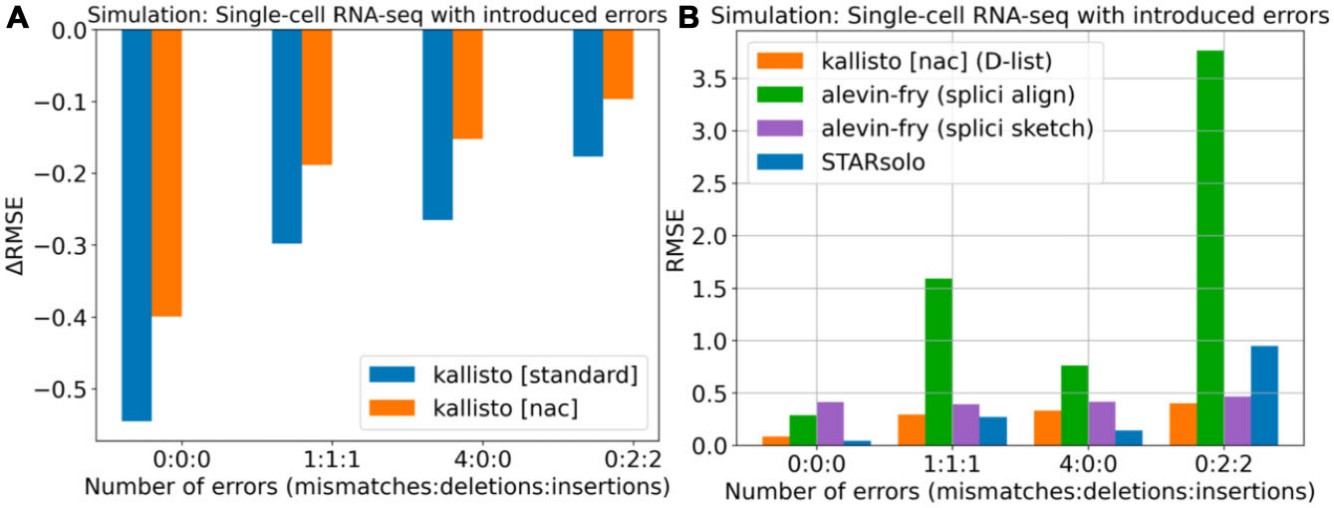
Assessment of different mapping modes on simulated data generated using the STARsolo simulation framework including the introduction of errors into the reads. (**A**) Reduction in quantification error, as measured by change in RMSE, by using a D-list to index DFKs compared with not using a D-list on simulated reads with mismatches, deletions and insertions. (**B**) Quantification error of different tools on simulated reads with mismatches, deletions and insertions. splici align: enabling the index used by alevin-fry that contains introns as well as selective alignment mode. splici sketch: Same as “splici align” except selective alignment mode is disabled. The errors were introduced into the “single-cell RNA-seq (no multigene)” simulated reads (see “Materials and methods” section).

Since DFKs improve mapping specificity, a natural question that arises is whether the improvement in accuracy scales with higher sequencing error rates. Particularly, how do DFKs compare to alignment-based approaches in maintaining accuracy in the face of more sequencing errors? To address this, we introduced additional sequencing errors, consisting of a combination of mismatches, insertions and deletions, into the STARsolo simulations (Supplementary Table S3). We found that the usage of DFKs always results in an improvement in accuracy, even with a high mismatch rate or a high indel rate within the simulated sequencing reads (Figure 7A). In contrast, while alignment-based methods tend to be robust to mismatch errors, they fall short with high indel rates (Figure 7B). In particular, the same selective alignment settings when executed on the original simulation and simulations where indels are introduced result in a substantial performance decrease on the indel simulations. Altogether, these results suggest that pseudoalignment with the incorporation of DFKs is more robust than alignment-based methods to indels. Such considerations may be important when mapping RNA-seq reads from technologies with higher indel rates, such as long-read RNA-seq (45,46).

### Nascent, mature and ambiguous classifications

**Figure 8.**
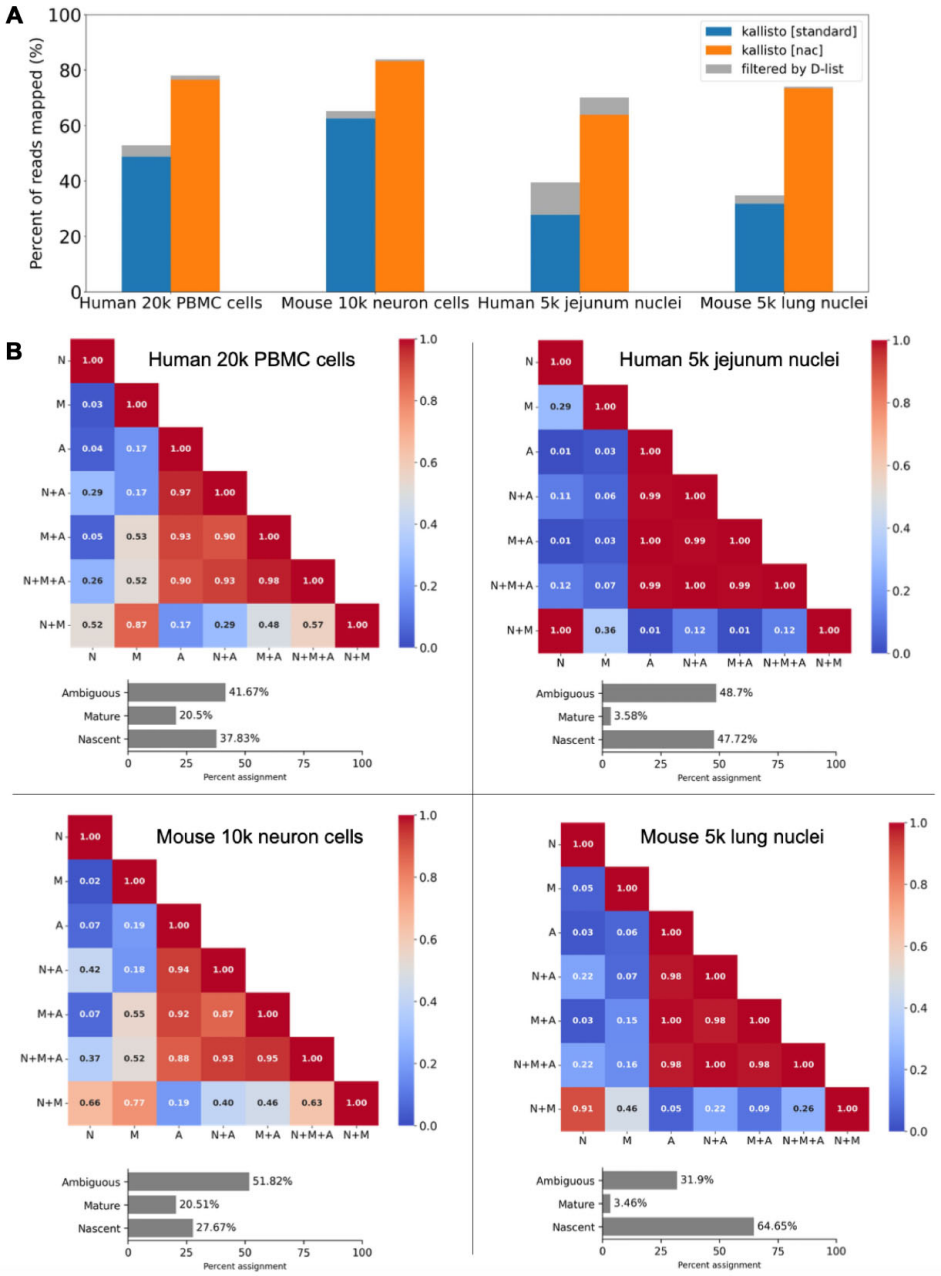
Quantification of mature and nascent RNA from single-cell and single-nucleus experiments. (**A**) The percentage of reads mapped by index type (standard or nac). The gray bar represents the reads that are excluded when the D-list is used. Of note, the standard index type only has mature and ambiguous transcripts in the index (M + A) while the nac index type has nascent, mature and ambiguous transcripts in the index (N + M + A). (**B**) Exploration of single-cell and single-nuclei count matrices from human and mouse samples (datasets from 10x Genomics). The heatmap shows the Pearson correlation coefficient between pseudo-bulked count matrices, produced by mapping RNA-seq reads against the nac index with the D-list. Pseudo-bulking was performed on cell barcodes with at least 500 UMIs detected in the N + M + A (total) matrix. The bar plots show the percentage of UMIs assigned to the ambiguous, nascent and mature classifications. N: Nascent, M: Mature, A: Ambiguous.

The extended transcriptome index (i.e. the nac index type), by virtue of indexing intron-containing nascent transcripts, enables the mapping of a substantial fraction of reads that would otherwise go unmapped when using the standard index type (Figure 8A). Furthermore, the quantifications produced by the nac index can classify reads or UMIs as mature (M), nascent (N) or ambiguous (A). We assessed the classifications on both single-cell and single-nucleus data from mouse and human samples. As expected, single-nucleus data tends to have a higher ratio of nascent to mature RNA compared to single-cell data since RNA molecules that have been exported out of the nucleus have undergone splicing and maturation (Figure 8B) while 10x Genomics Visium spatial transcriptomics data has the lowest proportion of nascent RNA (<1%) due to the Visium kit’s exon capture (Supplementary Figure S6). Across the different count matrices, we observe that the total counts (N + M + A) are well-correlated with the ambiguous counts, implying that the results of a single-cell or single-nucleus RNA-seq analyses are largely driven by reads mapped solely within exons. Note that regardless of assay type, there tend to be more UMIs classified as nascent than mature, because introns have a much larger coverage over the genome than exon–exon SJs. The individual N, M and A count matrices are poorly correlated with one another, reflecting that different information is present in each of those three matrices. As biophysical models of the RNA life cycle make use of nascent transcript counts and mature transcript counts, how to allocate those ambiguous counts to either nascent or mature remains a topic for future research. For now, one might reasonably assume that the ambiguous counts in single-cell RNA-seq experiments originate from mature transcripts since, in such assays, it is expected that there will be more mature transcripts than nascent transcripts therefore a purely-exonic UMI is most likely to be mature. However, in the nucleus context, the likelihood of a purely-exonic UMI being mature is lower since there will be fewer mature transcripts, as evidenced by the much larger nascent-to-mature ratio in UMI classification. Developing methods to more accurately allocate ambiguous reads is an interesting topic to pursue but is beyond the scope of this study.

**Figure 9.**
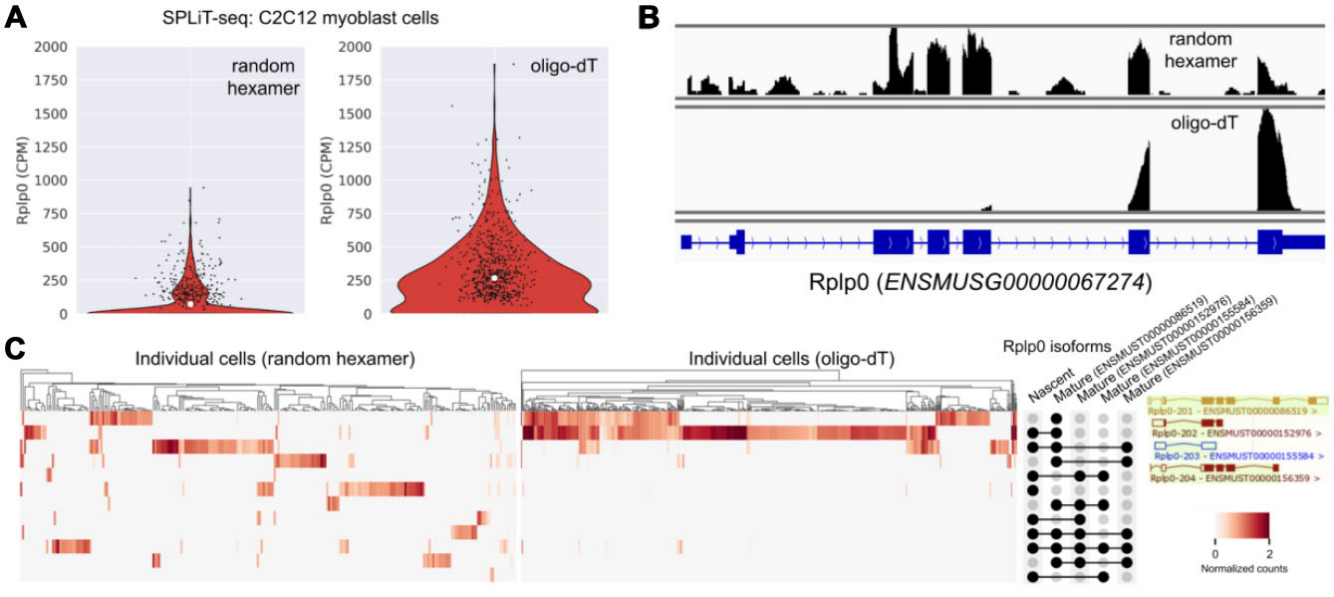
Isoform compatibility quantification of nascent and mature RNA. (**A**) Rplp0 gene-level counts (nascent + mature + ambiguous) of mouse C2C12 myoblast cells from a SPLiT-seq single-cell RNA-seq assay, wherein reads from the random hexamer priming strategy were quantified separately from the reads with the oligo-dT priming strategy. The Rplp0 CPMs (counts per million) of individual cells are plotted. (**B**) Genome browser tracks of the reads aligned to the Rplp0 gene. (**C**) Normalized TCCs of UMIs assigned to the Rplp0 gene. Each row in the heatmap represents an EC (i.e. a set of transcripts that a UMI is compatible with) and each column represents an individual cell. ECs essentially capture UMI assignment ambiguity between isoforms and, when mapping reads to the nac index, between nascent and mature status. The transcripts that constitute each of the 12 ECs shown are presented in the UpSet plot labels to the right of the heatmap. Each UMI within a given cell is assigned to an EC and a transcript isoform can be present in multiple ECs. The isoform structures shown on the right were obtained from ENSEMBL.

Moreover, mature RNA can have multiple isoforms and a comprehensive analysis will identify not only whether a UMI originated from mature RNA produced by a gene but also which mature RNA of that gene the UMI originated from. As pseudoalignment works by identifying a set of targets that a UMI is compatible with, it is straightforward to determine whether those set of targets contain specific isoforms of a gene. To investigate how this may potentially be useful, we utilized SPLiT-seq (47) data of mouse myoblasts (27). We chose to analyze SPLiT-seq data because, in that technology, the same cell can be sequenced using an oligo-dT priming strategy and a random hexamer priming strategy. These two priming strategies will yield different isoform abundances as the oligo-dT primer selects for the polyA tail of mRNA while the random hexamer does not. An example from the gene Rplp0, which is present in both the oligo-dT library and the random hexamer library, albeit to a lesser extent in the latter, illustrates the difference (Figure 9A). The random hexamer reads cover the entire gene body, including in intronic regions, while the oligo-dT reads are heavily localized to the 3′ end region of the gene with very few reads elsewhere or in introns (Figure 9B). Upon using the nac index to resolve nascent and isoform-level mature RNA (Figure 9C), we find that a large number of sequenced molecules from the oligo-dT library are of mature status and, specifically, belong to isoform ENSMUST00000086519, which is a transcript that extends to the 3′ end of the gene (48). Nascent RNA and other isoforms primarily originate from the random hexamer library. Although this analysis was only done at the target-compatibility level (including both nascent and mature RNA as targets in contrast to previous approaches which only included mature RNA), one can use such TCCs directly in cell clustering analysis (49,50). While an expectation-maximization algorithm can attempt to probabilistically assign TCCs to transcript-level estimates (Supplementary Figure S7), an identifiability problem due to a high degree of ambiguity may preclude robust estimates from being obtained when quantification relies on short reads. Finally, while some work has been done in jointly using nascent and mature RNA counts, as produced by kallisto for biophysically motivated cell clustering analysis (51), utilizing isoform-level mature RNA to further enhance such analysis is an avenue for future research (52).

### The effects of different index strategies on single-cell clustering and marker gene selection

**Figure 10.**
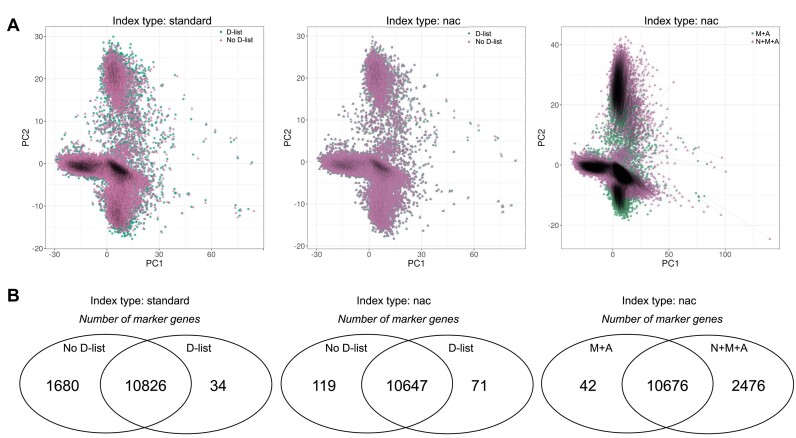
Effect of index strategy and count matrix type on single-cell RNA-seq analysis. (**A**) PCA of cells from the human 20k PBMC dataset (10x Genomics) for count matrices generated in various ways. The first two principal components are shown. Black lines connect identical cell barcodes from each matrix used in pairwise comparisons. (**B**) Number of marker genes identified through differential gene expression analysis for count matrices generated in various ways. Left-hand panel: Count matrices were generated by mapping reads to the standard index type with and without a D-list. Middle panel: Count matrices (M + A) were generated by mapping reads to the nac index type with and without a D-list. Right panel: M + A and N + M + A count matrices were generated by mapping reads to the nac index type. N: nascent, M: nature and A: ambiguous.

We explored how different index types and different count matrices may affect downstream clustering analysis and marker gene selection using the approach of (35). Using the human 20k PBMC dataset (10x Genomics), we projected filtered count matrices (high-quality cells and highly variable genes) onto the first two principal components through PCA (Figure 10A). Applying a D-list to the standard index type affected cell projections, but the impact was mild, likely because the human reference genome is a comprehensive and well-annotated assembly, reducing the chance of reads from exonic regions being mismapped. The effect was even more subtle when applying a D-list to the nac index, which includes intronic regions. A more significant difference emerged when comparing analyses with and without nascent transcript quantification. When identifying marker genes through differential gene expression, application of the D-list led to a fraction of marker genes (2% for the standard index type; 14% for the nac index) being uniquely identified in one condition (D-list or no D-list) but not the other, while incorporating nascent transcript quantification resulted in 19% more marker genes being identified (Figure 10B). Although, broadly, cluster analysis remained largely unaffected by these different strategies (Supplementary Figure S8), the simulations earlier on in this paper showed that more pronounced differences can be observed at the individual cell and gene level, thus making the selection of index strategy an important consideration depending on the type of downstream analysis that is to be performed. Additionally, “mature” and “nascent” quantifications provide distinct insights into the cell’s profile. Although count matrices containing only “nascent” or “mature” gene counts can sometimes yield similar clusters, the cellular profile differs greatly, with many marker genes unique to each matrix (Supplementary Figure S9).

## Discussion

**Figure 11.**
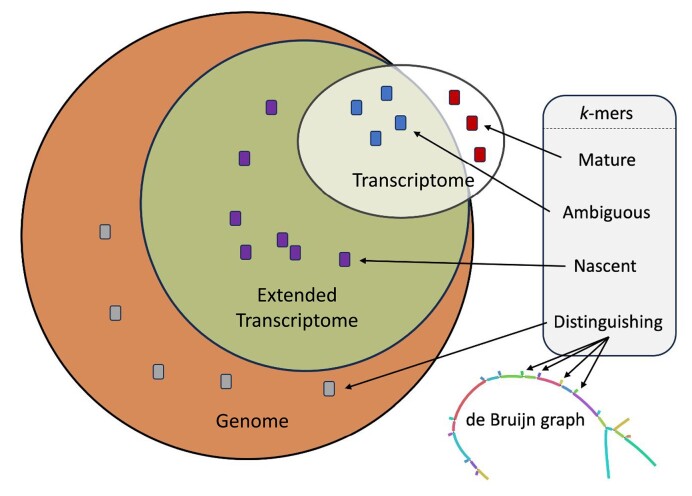
Summary of enhancements to read mapping and classification. This figure shows *k*-mers originating from the standard transcriptome index, the extended transcriptome index containing nascent RNA transcripts, and the entire genome. The integration of DFKs into a dBG is shown.

This study introduces a combined approach of using DFKs with an extended transcriptome index (nac index) in single-cell and single-nucleus RNA-seq analysis (Figure 11). This method aims to address specific challenges in RNA-seq, particularly in the quantification of nascent and mature mRNA transcripts, and in reducing mismapping errors caused by reads originating outside of the targeted transcriptomic regions.

While this method can provide accurate quantification of mature RNA transcripts, nascent RNA transcripts and ambiguous RNA transcripts (i.e. transcripts that cannot be unambiguously resolved as nascent or mature), how to jointly utilize these three types of RNA transcripts remains an avenue for future research. One approach to “integrating” the nascent and mature modalities is via biophysical modeling of transcription (3,10,11); however questions remain, such as how to best utilize reads that are ambiguous between the modalities. Importantly, there is not one single “count matrix”; rather, there are multiple count matrices that each lend value in single-cell and single-nucleus RNA-seq analyses. The number of count matrices becomes even larger when considering technologies such as SPLiT-seq (47), for which two different priming strategies (oligo-dT and random hexamer) exist for a single cell, or Smart-seq3 (53), for which two complementary DNA (cDNA) fragment types (UMI and internal) exist, thus resulting in an additional set of count matrices (Supplementary Figure S10). The ability to differentiate and quantify nascent, mature and ambiguous transcripts offers a more nuanced view of gene expression, potentially enriching our understanding of RNA processing and transcriptome dynamics.

There are several limitations to the quantification framework we have proposed. In a cell, the set of unprocessed mRNAs at any given time is likely to include partially processed molecules (54,55), and in principle the complete splicing cascade must be understood and known in order to accurately quantify single-nucleus or single-cell RNA-seq data (52). Furthermore, the presence of ambiguous reads both for single-cell and single-nucleus RNA-seq is unsatisfactory. Ideally reads should be longer so that they can be uniquely classified, or they should be fractionally classified probabilistically. The latter approach is nontrivial due to variation in effective transcript lengths that will depend on library preparation and must be accounted for (56,57), but this is an interesting direction of study.

While most mismapping errors that affect single-cell RNA-seq quantification are eliminated by extending the transcriptome index, DFKs provide further improvement to quantification accuracy. Specifically, DFKs can eliminate erroneous mapping of reads that originate from transcripts that appear outside even the extended transcriptome index. More importantly, DFKs provide high scalability. DFKs can scale to higher sequencing error rates as the accuracy gains of DFKs are not reversed when different sequencing error profiles are introduced. Moreover, DFKs can scale to size. When only a small specific set of targets is of interest but there are many known possible target sequences, those possible target sequences can simply be incorporated into the D-list. The resultant DFKs will optimize mapping specificity making it unnecessary to index all the possible target sequences. Irrespective of whether the target sequences occupy a small proportion or a large proportion of the “background”, the DFKs will improve mapping specificity without any major impact on performance. Thus, the DFKs act as a space-efficient general “background filter”.

In summary, this study introduces a method for improving the accuracy of generating count matrices. It is anticipated that these improved quantifications and the multimodal nature of these quantifications will prove useful for multiple downstream applications, including both total gene expression quantification and the integration of multiple count matrices via biophysically informed models.

## Supplementary Material

gkae1137_Supplemental_File

## Data Availability

The human reference genome (GRCh38) used throughout is the same one used in the STARsolo simulations (9). The GRCh38 FASTA and GTF files used are available from the code repository associated with this paper. The mouse reference genome (GRCm39) used is the primary assembly FASTA file from Ensembl with the corresponding GTF annotation version 110, which was filtered to only include the gene_biotype values of protein_coding, lncRNA, lincRNA and antisense. The following sequencing datasets, publicly available from 10x Genomics (all version 3 chemistry except one Visium technology dataset), were used: Human 5k PBMC (single cell) Sample name: 5k_pbmc_v3 383 941 607 reads Human 20k PBMC (single cell) Sample name: 20k_PBMC_3p_HT_nextgem_Chromium_X) 818 107 363 reads Human 5k jejunum (single nucleus) Sample name: 5k_human_jejunum_CNIK_3pv3 121 378 620 reads Mouse 10k neuron (single cell) Sample name: SC3_v3_NextGem_SI_Neuron_10K 1 589 915 447 reads Mouse 5k lung (single nucleus) Sample name: 5k_mouse_lung_CNIK_3pv3) 232 479 932 reads Mouse embryo Visium CytAssist 11mm FFPE Sample name: CytAssist_11mm_FFPE_Mouse_Embryo 832 193 962 reads The datasets can be downloaded from https://www.10xgenomics.com/. Additionally, the mouse SPLiT-seq data (27) analyzed in this study can be found at GEO accession identifier GSE168776; all seven short read sequencing subpools within that dataset were used. This study did not produce or analyze any other datasets.
