# Supplementary material for: Accurate quantification of nascent and mature RNAs from single-cell and single-nucleus RNA-seq
Source: Nucleic Acids Res. 2024 Dec 6;53(1):gkae1137. doi: 10.1093/nar/gkae1137 (PMC11724275; doi:10.1093/nar/gkae1137)
Supplement: gkae1137_Supplemental_File [file gkae1137_supplemental_file.pdf]

Supplementary Information

Accurate quantification of nascent and mature RNAs from single-cell and single-nucleus RNA-seq

Delaney K. Sullivan[1,2#], Kristján Eldjárn Hjörleifsson[3#], Nikhila P. Swarna[1], Conrad Oakes[1], Guillaume Holley[4], Páll Melsted[4,5*], Lior Pachter[1,3*]

Supplementary Figure 1: The maturation process of RNA transcripts.

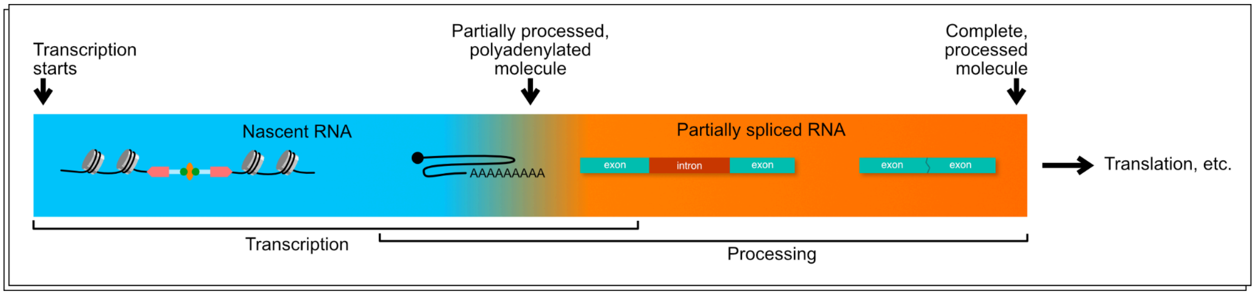

Supplementary Figure 2: Performance comparison of different implementations of the nac index type.

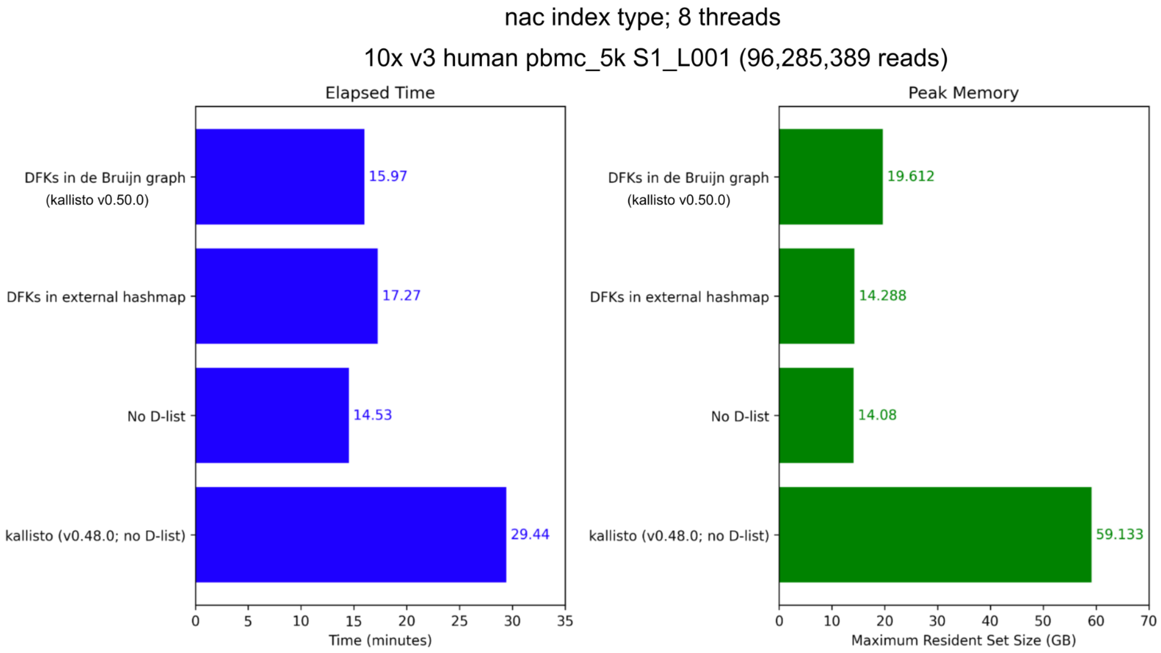

Supplementary Figure 3: Improvement with the D-list on kallisto transcript-level quantification results, assessed using the standard index type with an incomplete transcriptome on simulated bulk RNA-seq reads.

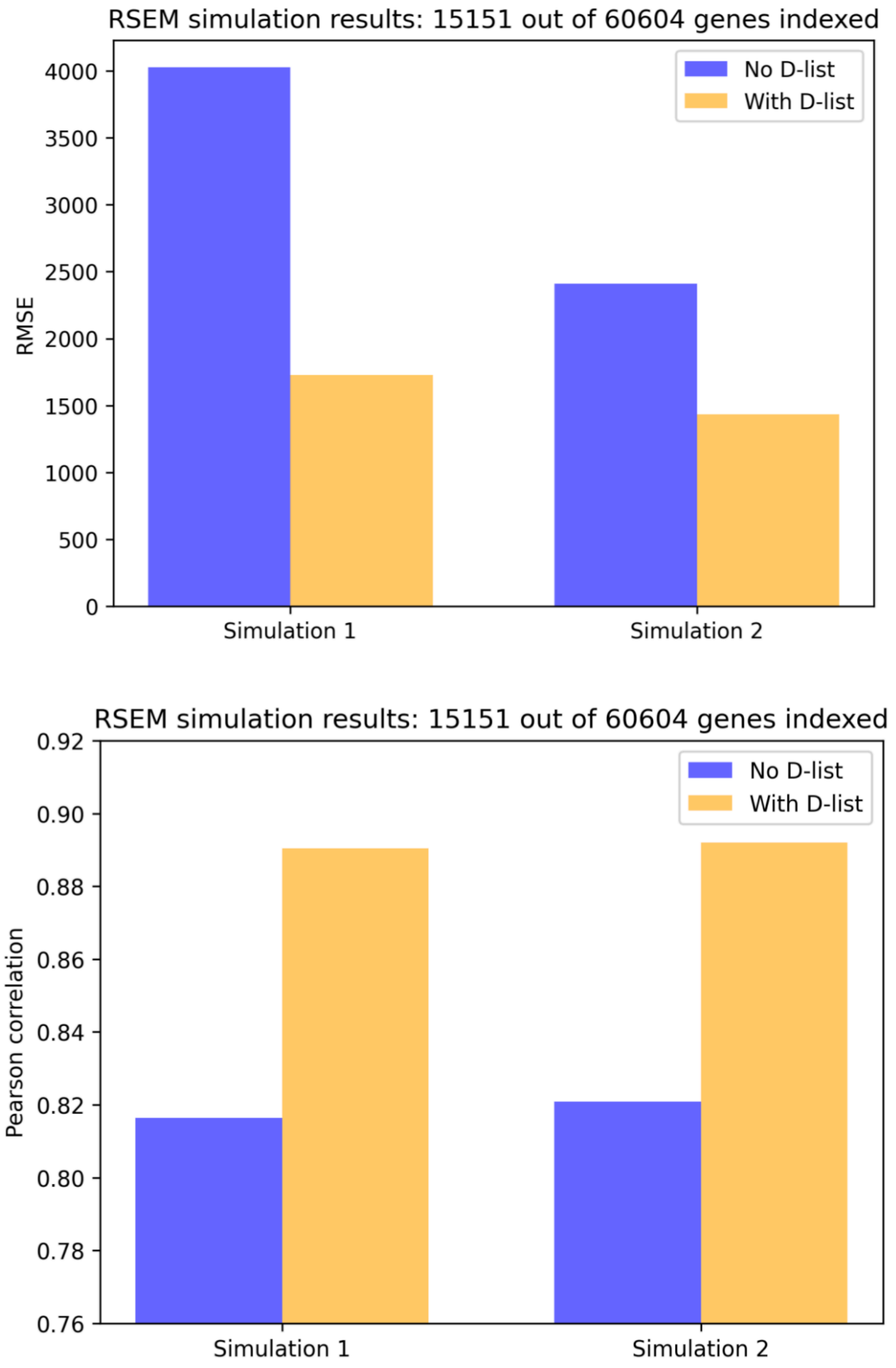

Supplementary Figure 4: Assessment of kallisto quantification results, without multimapping mode enabled, on simulated single-cell RNA-seq reads that include multi-gene reads.

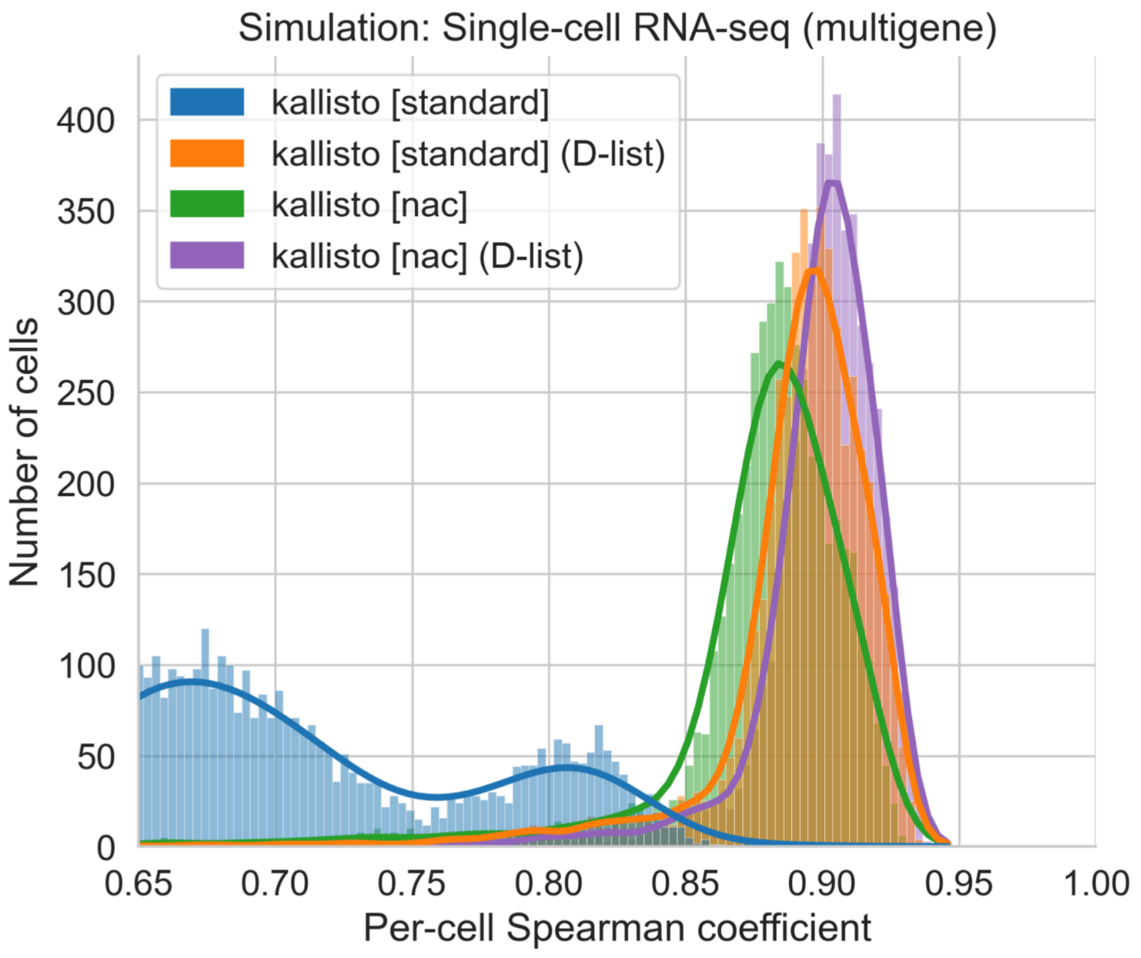

Index type	D-list	mult	Median ρ*	Median r	RMSE	FPR	FNR
standard			0.679939	0.991087	0.682919	0.019123	0.002950
standard	✓		0.896340	0.997867	0.287012	0.000783	0.003218
nac			0.883905	0.994031	0.555755	0.000626	0.003342
nac	✓		0.902738	0.997835	0.289252	0.000197	0.003367

Supplementary Figure 5: Assessment of kallisto quantification results with multimapping mode enabled on simulated single-cell RNA-seq reads that do not include multi-gene reads.

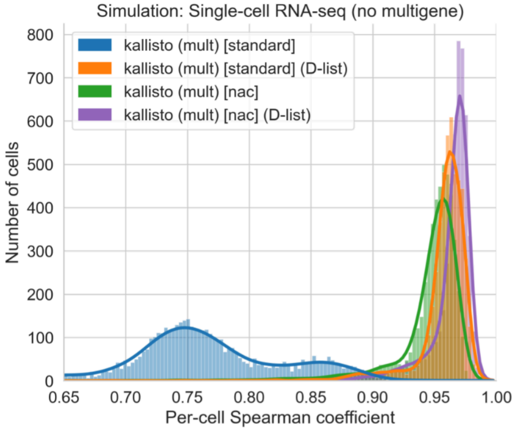

Index type	D-list	mult	Median ρ*	Median r	RMSE	FPR	FNR
standard		✓	0.756161	0.992337	0.653089	0.050188	0.000241
standard	✓	✓	0.961758	0.999728	0.104715	0.002396	0.000431
nac		✓	0.953292	0.995827	0.506452	0.023166	0.000517
nac	✓	✓	0.968557	0.999734	0.105673	0.018768	0.000542

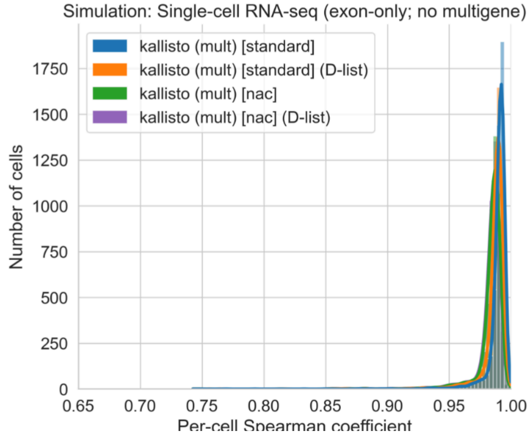

Index type	D-list	mult	Median ρ*	Median r	RMSE	FPR	FNR
standard		✓	0.991460	0.999955	0.039947	0.000249	0.000321
standard	✓	✓	0.989052	0.999925	0.050895	0.000173	0.000448
nac		✓	0.986388	0.999891	0.061643	0.000508	0.000554
nac	✓	✓	0.985920	0.999878	0.065441	0.000473	0.000575

Supplementary Figure 6: The different count matrices and their combinations produced from the mouse embryo Visium CytAssist 11mm FFPE spatial transcriptomics dataset with 832,193,962 reads.

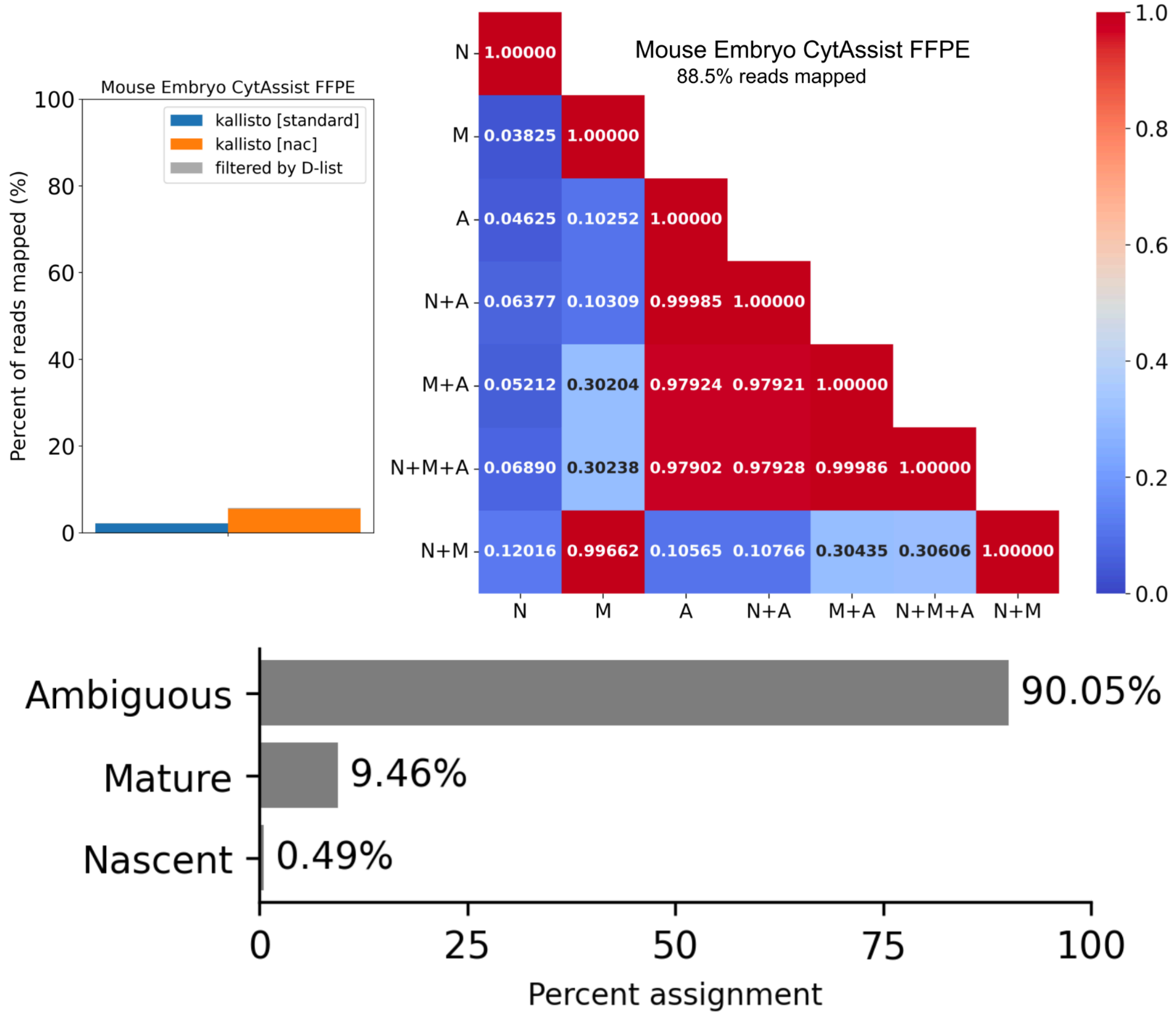

Supplementary Figure 7: Example of the results of expectation-maximization probabilistic assignment of UMIs to nascent and mature transcripts in SPLiT-seq data with the barcodes from the two priming strategies collapsed together.

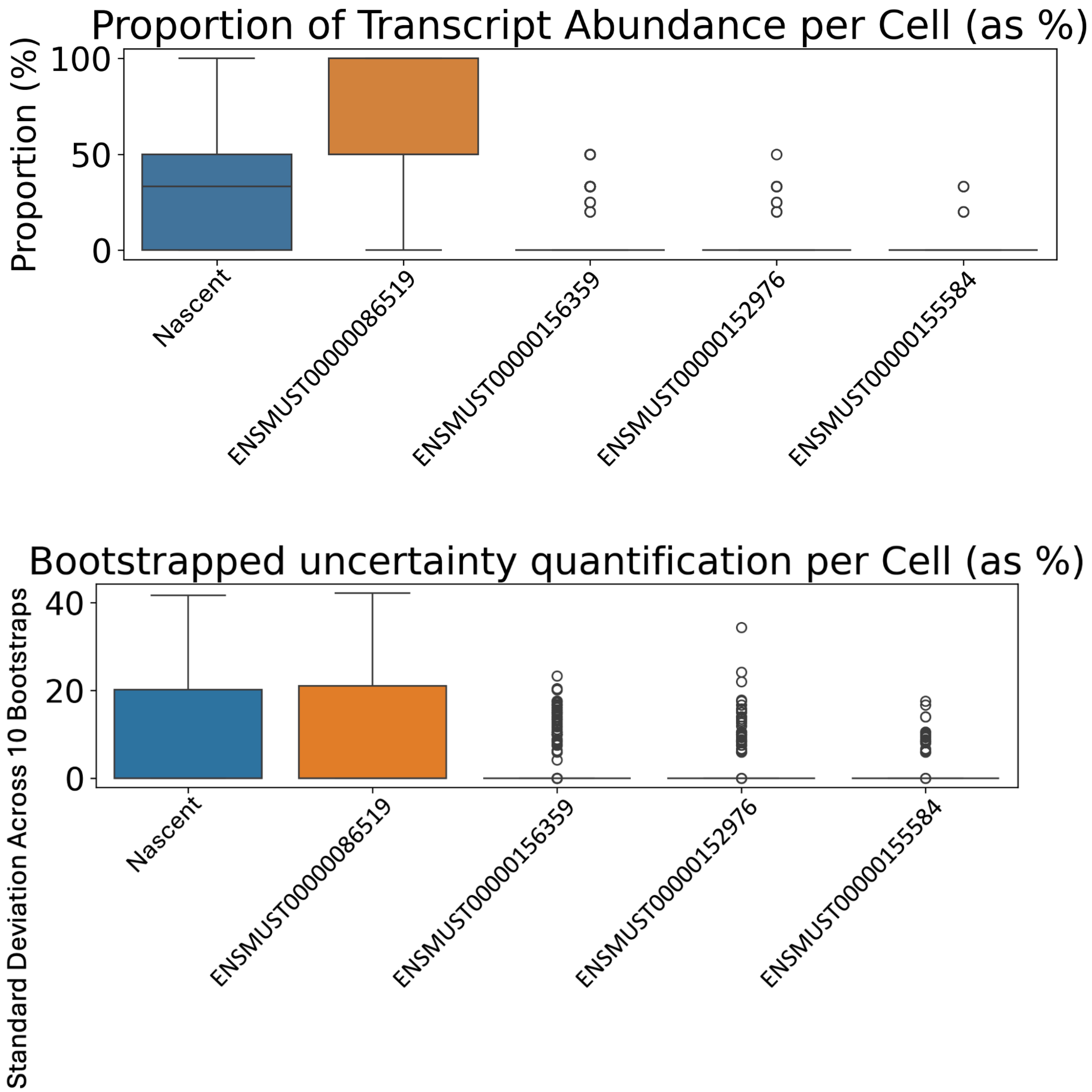

Supplementary Figure 8: Alluvial plots of cluster assignment mapping and UMAP plots for pairwise comparisons of count matrices generated by different index strategies in human 20k PBMC single-cell RNA-seq data.

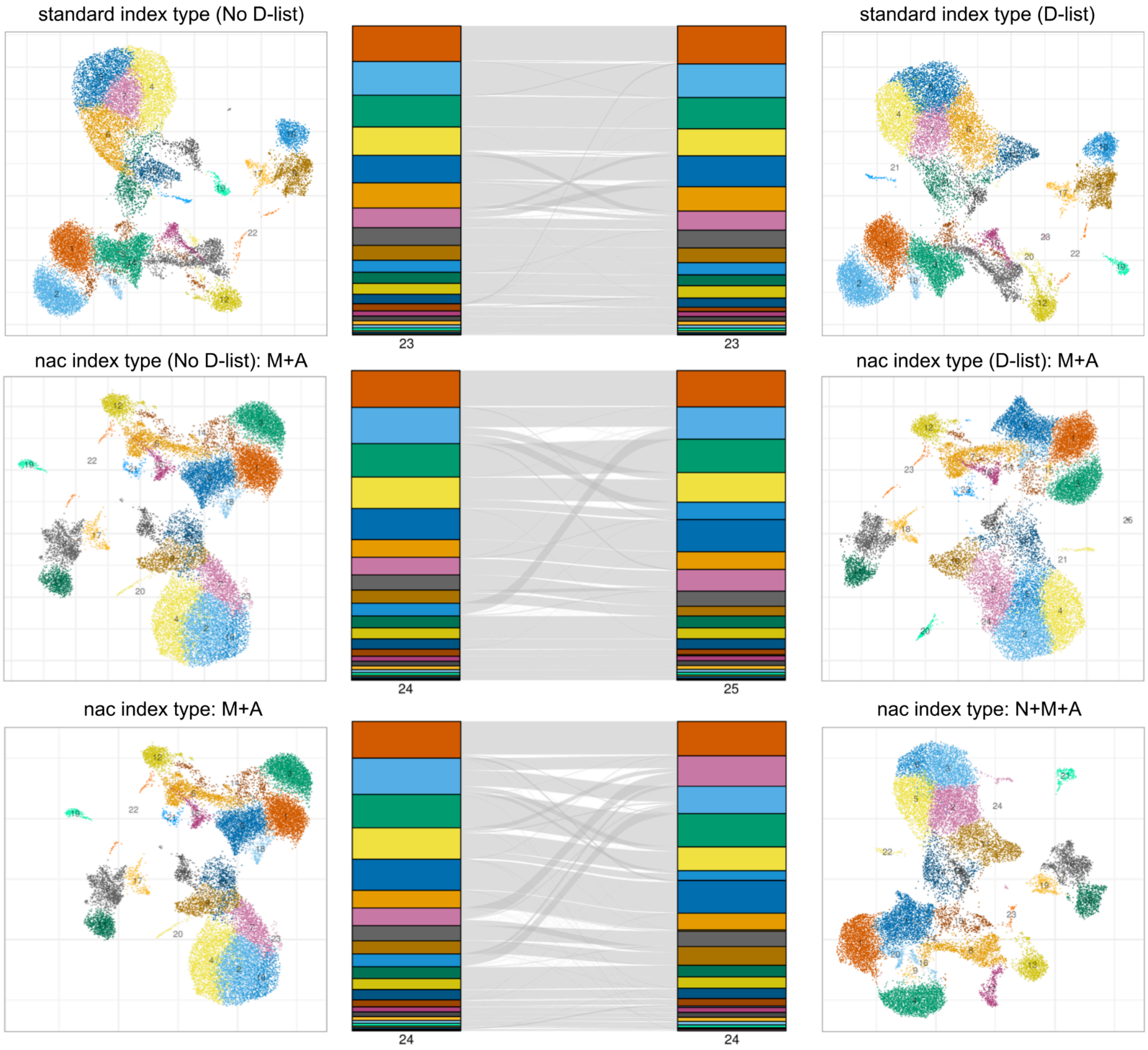

Supplementary Figure 9: Alluvial plots of cluster assignment mapping, marker gene numbers, PCA plots, and UMAP plots for comparing nascent and mature count matrices in human 20k PBMC single-cell RNA-seq data.

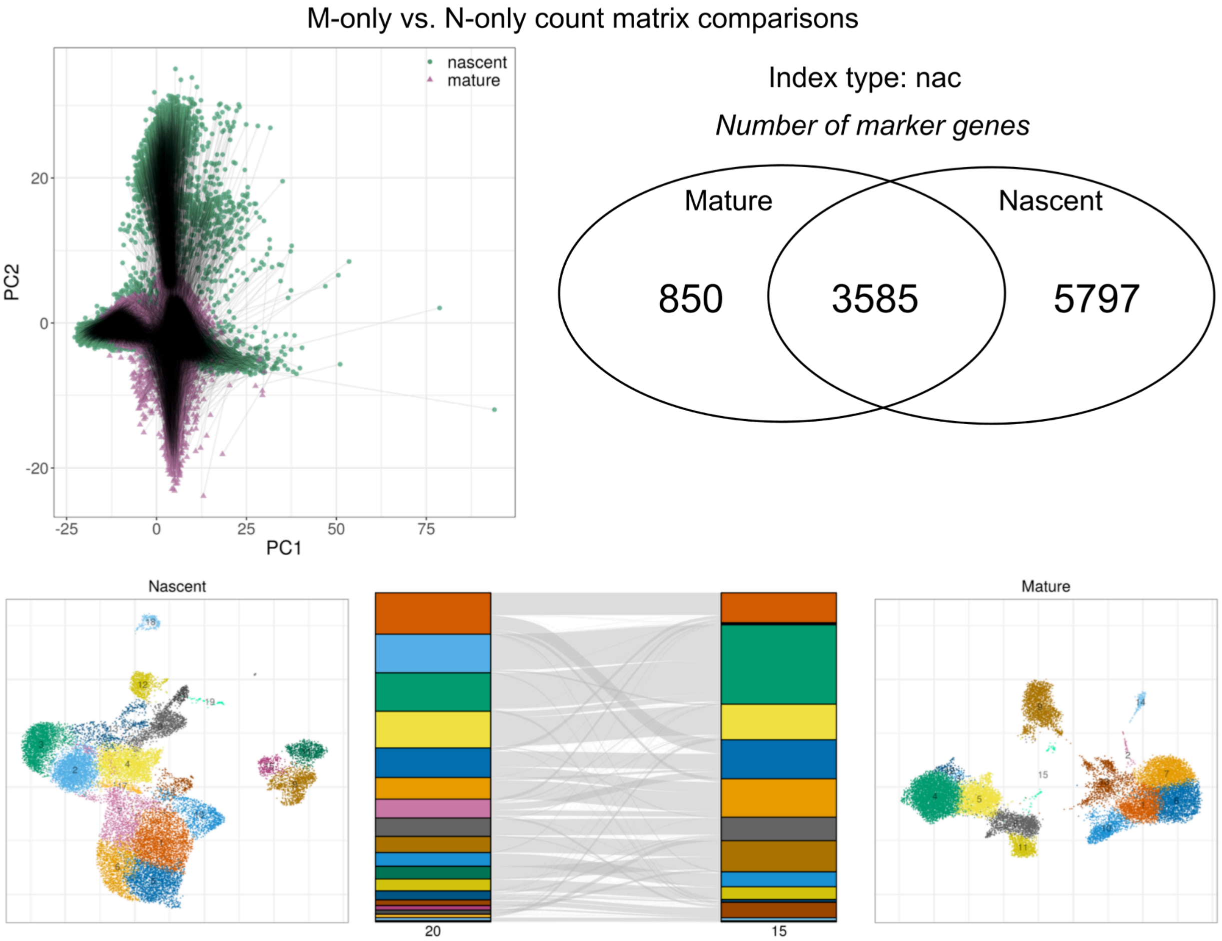

Supplementary Figure 10: Depiction of the distinct count matrices that can be studied with data from a single-cell or single-nucleus genomics experiment.

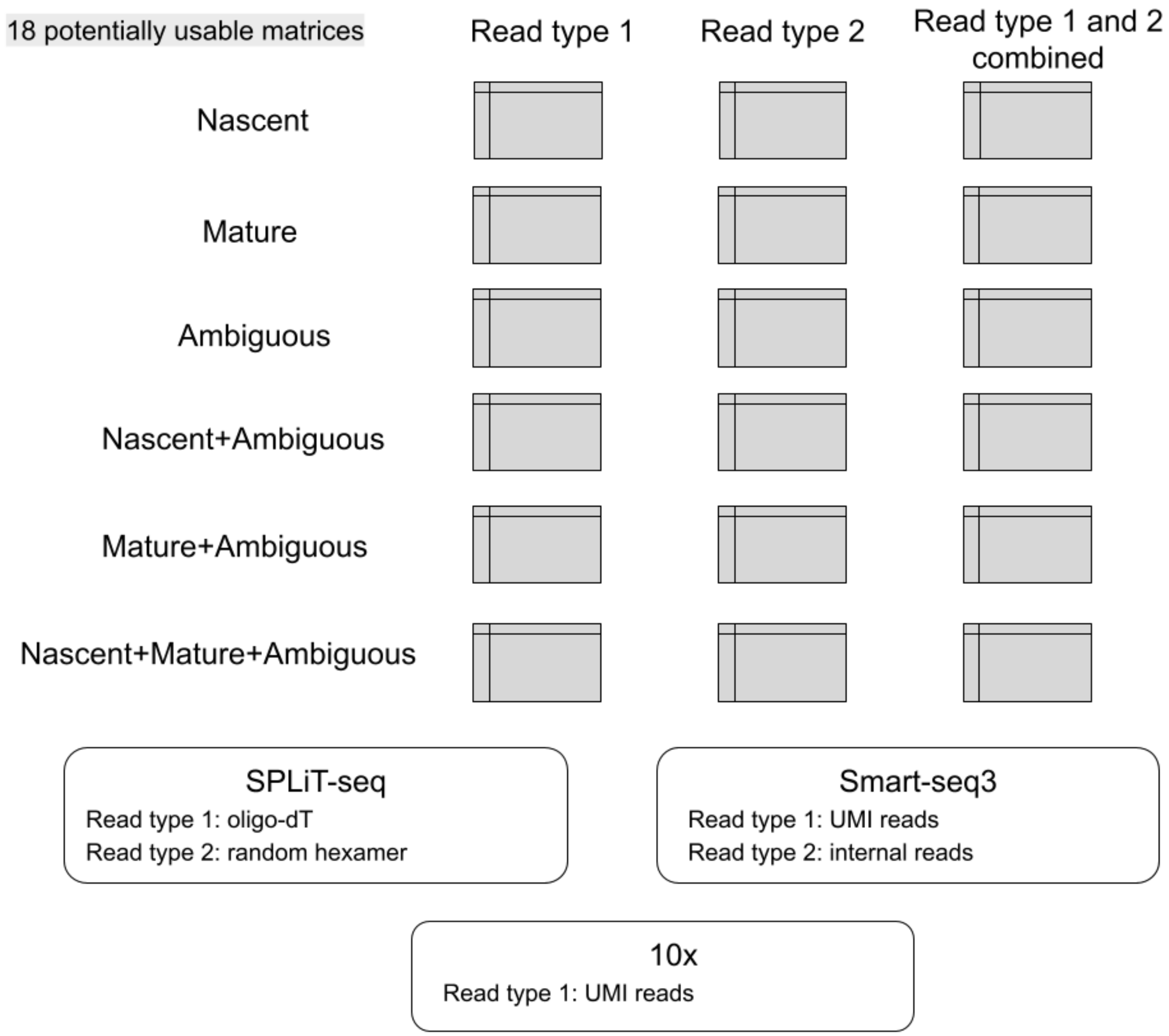

Supplementary Table 1: Evaluation metrics of kallisto on simulated data as a function of DFK overhang length.

**Index type**	**Overhang**	**mult**	**Median ρ***	**Median r**	**RMSE**	**FPR**	**FNR**	***k*-mers**	**DFKs**
Simulation: Single-cell RNA-seq (no multigene)									
standard	2		0.973984	0.999819	0.080112	0.000739	0.000495	113,209,587	9,649,025
standard	3		0.974748	0.999824	0.078881	0.000685	0.000506	113,209,587	15,696,903
standard	4		0.97535	0.999826	0.07816	0.000643	0.000513	113,209,587	22,293,140
standard	5		0.975842	0.99983	0.077472	0.000608	0.000519	113,209,587	29,278,838
standard	6		0.976265	0.999833	0.076833	0.00058	0.000524	113,209,587	36,550,263
nac	2		0.980336	0.999812	0.083905	0.000202	0.000618	1,398,470,117	21,338,179
nac	3		0.980491	0.999814	0.08297	0.000197	0.000619	1,398,470,117	30,833,829
nac	4		0.980585	0.999816	0.082474	0.000193	0.00062	1,398,470,117	39,760,674
nac	5		0.980698	0.999817	0.082168	0.000189	0.000621	1,398,470,117	48,233,865
nac	6		0.980771	0.999819	0.081806	0.000185	0.000622	1,398,470,117	56,344,346

mult: the multimapping quantification mode is enabled (not enabled for any of the runs here). Overhang: The number of *k*-mers flanking a unitig to be considered a DFK. ρ*: Modified spearman correlation. r: Pearson correlation. RMSE: root mean squared error. FPR: false positive representation. FNR: false negative representation. See **Methods** for details.

Supplementary Table 2: Evaluation of the STARsolo, Cell Ranger, alevin-fry and cellCounts single-cell RNA-seq programs on simulated data generated using the STARsolo simulation framework.

**Program**	**Run mode**	**Median ρ***	**Median r**	**RMSE**	**FPR**	**FNR**
Simulation: Single-cell RNA-seq (exon-only; no multigene)						
STARsolo		0.993564	0.999968	0.030486	0.000053	0.000270
Cell Ranger	--include-introns=false	0.956866	0.999547	0.232195	0.000030	0.000343
alevin-fry	splici align	0.984427	0.999902	0.069541	0.000030	0.000709
alevin-fry	splici sketch	0.982900	0.999889	0.062949	0.000031	0.000743
alevin-fry	align	0.990880	0.999931	0.060543	0.000030	0.000466
alevin-fry	sketch	0.991528	0.999948	0.043601	0.000031	0.000393
cellCounts		0.915796	0.994174	0.521436	0.000239	0.001337
Simulation: Single-cell RNA-seq (no multigene)						
STARsolo		0.991877	0.999940	0.042743	0.000140	0.000270
Cell Ranger	--include-introns=false	0.955377	0.999499	0.232933	0.000081	0.000343
alevin-fry	splici align	0.971626	0.998672	0.288308	0.000314	0.000702
alevin-fry	splici sketch	0.960025	0.997419	0.414070	0.000764	0.000739
alevin-fry	align	0.879684	0.998145	0.312199	0.005676	0.000446
alevin-fry	sketch	0.778933	0.995372	0.527728	0.016063	0.000357
cellCounts		0.825302	0.993000	0.532384	0.005890	0.001299

Modified spearman correlation. r: Pearson correlation. RMSE: root mean squared error. FPR: false positive representation. FNR: false negative representation. See **Methods** for details.

alevin-fry options:

- splici align: Enabling the index used by alevin-fry that contains introns as well as selective alignment mode.
- splici sketch: Enabling the index used by alevin-fry that contains introns without selective alignment mode.
- align: Selective alignment enabled and index is a standard transcriptome index that does not include introns in alevin-fry.
- sketch: Selective alignment disabled and index is a standard transcriptome index that does not include introns in alevin-fry.

For Cell Ranger, version 7 was used with the include-introns option set to false in order to mimic the default behavior of versions 1,2,3,4,5 and 6.

Supplementary Table 3: Evaluation metrics of the kallisto, alevin-fry, and STARsolo single-cell RNA-seq programs on simulated data generated using the STARsolo simulation framework with errors introduced into sequencing reads.

Mismatches	Deletions	Insertions	Program	Run mode	Median ρ*	Median r	RMSE	FPR	FNR
Simulation: Single-cell RNA-seq (no multigene)									
1	1	1	kallisto	standard (no D-list)	0.629277	0.993046	0.575156	0.030304	0.001565
1	1	1	kallisto	standard (D-list)	0.87452	0.998388	0.276873	0.003689	0.002376
1	1	1	kallisto	nac (no D-list)	0.890008	0.995764	0.484241	0.001393	0.002872
1	1	1	kallisto	nac (D-list)	0.900935	0.998259	0.29544	0.001023	0.002973
1	1	1	alevin-fry	splici align	0.673962	0.989232	1.592164	0.000077	0.018216
1	1	1	alevin-fry	splici sketch	0.899393	0.99736	0.39301	0.001391	0.002823
1	1	1	STARsolo		0.911674	0.998962	0.271059	0.000888	0.002918
4	0	0	kallisto	standard (no D-list)	0.612826	0.992954	0.570622	0.030607	0.002184
4	0	0	kallisto	standard (D-list)	0.85271	0.998136	0.305361	0.004151	0.002991
4	0	0	kallisto	nac (no D-list)	0.870739	0.995728	0.483343	0.001446	0.003621
4	0	0	kallisto	nac (D-list)	0.881683	0.997985	0.330969	0.001098	0.003706
4	0	0	alevin-fry	splici align	0.819814	0.996343	0.76258	0.000141	0.008301
4	0	0	alevin-fry	splici sketch	0.878774	0.9971	0.415686	0.00136	0.003727
4	0	0	STARsolo		0.948253	0.999501	0.141767	0.000947	0.001244
0	2	2	kallisto	standard (no D-list)	0.586625	0.993183	0.557911	0.030393	0.002935
0	2	2	kallisto	standard (D-list)	0.831982	0.997594	0.381203	0.00433	0.003804
0	2	2	kallisto	nac (no D-list)	0.851074	0.99581	0.500121	0.001891	0.004287
0	2	2	kallisto	nac (D-list)	0.861993	0.997502	0.403226	0.001458	0.004384
0	2	2	alevin-fry	splici align	0.412969	0.924791	3.764902	0.000033	0.053289
0	2	2	alevin-fry	splici sketch	0.852768	0.996873	0.466828	0.00178	0.004671
0	2	2	STARsolo		0.763642	0.994432	0.948656	0.000552	0.010949

Modified spearman correlation. r: Pearson correlation. RMSE: root mean squared error.
FPR: false positive representation. FNR: false negative representation. See **Methods** for details.
alevin-fry options:

- splici align: Enabling the index used by alevin-fry that contains introns as well as selective alignment mode.
- splici sketch: Enabling the index used by alevin-fry that contains introns without selective alignment mode.
